# 3D Pose Estimation and Tracking in Handball Actions Using a Monocular Camera

**DOI:** 10.3390/jimaging8110308

**Published:** 2022-11-10

**Authors:** Romeo Šajina, Marina Ivašić-Kos

**Affiliations:** 1 Faculty of Informatics, University of Pula, 52100 Pula, Croatia; 2 Faculty of Informatics and Digital technologies, University of Rijeka, 51000 Rijeka, Croatia; 3 Centre for Artificial Intelligence, University of Rijeka, 51000 Rijeka, Croatia

**Keywords:** pose estimation, pose retargeting, sequence smoothing, tracking, deep learning models

## Abstract

Player pose estimation is particularly important for sports because it provides more accurate monitoring of athlete movements and performance, recognition of player actions, analysis of techniques, and evaluation of action execution accuracy. All of these tasks are extremely demanding and challenging in sports that involve rapid movements of athletes with inconsistent speed and position changes, at varying distances from the camera with frequent occlusions, especially in team sports when there are more players on the field. A prerequisite for recognizing the player’s actions on the video footage and comparing their poses during the execution of an action is the detection of the player’s pose in each element of an action or technique. First, a 2D pose of the player is determined in each video frame, and converted into a 3D pose, then using the tracking method all the player poses are grouped into a sequence to construct a series of elements of a particular action. Considering that action recognition and comparison depend significantly on the accuracy of the methods used to estimate and track player pose in real-world conditions, the paper provides an overview and analysis of the methods that can be used for player pose estimation and tracking using a monocular camera, along with evaluation metrics on the example of handball scenarios. We have evaluated the applicability and robustness of 12 selected 2-stage deep learning methods for 3D pose estimation on a public and a custom dataset of handball jump shots for which they have not been trained and where never-before-seen poses may occur. Furthermore, this paper proposes methods for retargeting and smoothing the 3D sequence of poses that have experimentally shown a performance improvement for all tested models. Additionally, we evaluated the applicability and robustness of five state-of-the-art tracking methods on a public and a custom dataset of a handball training recorded with a monocular camera. The paper ends with a discussion apostrophizing the shortcomings of the pose estimation and tracking methods, reflected in the problems of locating key skeletal points and generating poses that do not follow possible human structures, which consequently reduces the overall accuracy of action recognition.

## 1. Introduction

Human Pose Estimation (HPE) is a subfield of computer vision that aims to recognise the joints and skeleton of the human body in an image or video so that, based on these keypoints, a person’s position and orientation can be analysed, movements can be monitored and compared, motion and positions can be tracked, and various insights into the person’s activities can be drawn. It is a rapidly growing research area that has applications in various industries, including sports, dance, computer gaming, and healthcare. Some common use cases include action recognition and tracking, augmented reality experiences, animation, gaming, etc.

Today, almost all sports, both professional and recreational, rely heavily on data analytics and monitoring of athletes’ performance using various sensors and cameras in cell phone applications, smartwatches, and other human performance monitoring devices. As a result of an athletic activity, large amounts of recorded material are generated, which must be analysed to be useful. Analysing the material, especially the performance and movement of each athlete, is a tedious task that requires the expertise of kinesiologists, physical therapists, and sports experts, as well as many resources, and is therefore available only to clubs and high-level athletes.

The use of pose estimation methods can facilitate and speed up the process of analysing the athletes’ performance, especially in monitoring their movements, comparing techniques, and evaluating the proper execution of activities, and it can be made available to young athletes, small clubs, and recreational players. However, for automatic pose detection to be useful and usable in maintaining and improving physical activity and achieving the desired fitness, it must achieve high accuracy under real-world conditions and operate in real time. Useful and promising results in body posture detection and estimation were achieved in the era of Deep Learning, when deep convolutional neural networks were used to estimate the positions of keypoints on the body. One of the first deep neural networks that achieved promising results in human pose detection was DeepPose [1], which showed that deep neural networks could model invisible joints and perform much better under non-ideal conditions with occlusions. These results reversed the trend and paved the way for further research relying primarily on deep neural networks for pose estimation. Currently, the best results with deep learning models are obtained for individual sports and stationary exercises such as yoga or Pilates. However, the goal is to learn models for more complex scenarios, including more complex actions, non-standard poses, players, and team sports.

However, to analyse the execution of an action, in most cases one image or one frame is not sufficient, but it is necessary to track the person and his activity over a certain time sequence and a series of frames. The case where multiple objects appear on a scene that is observed over a period of time, taking into account the change in position of each object in the video sequence, is called multiple object tracking (MOT). Tracking provides the best results when the objects move uniformly, in the same direction, and without occlusion. However, this is usually not a realistic scenario, especially in complex scenes such as sporting events where a large number of players are being tracked, moving rapidly, changing their direction and speed, as well as their position and distance from the camera and the activity they are performing. In such dynamic scenes, tracking multiple objects remains a major challenge. However, thanks to improved object and skeleton detectors and computer power, pose tracking with object detection has become the leading paradigm for MOT.

In this paper, we present the current state of research on HPE based on Deep Learning, which can be useful for position estimation, tracking, action recognition, and action comparison of players in a dynamic team sport such as handball. First, we analyzed and compared related research that deals with tracking methods for observing and analyzing the motion of individuals based on the skeleton as a representation of a person. We also provide a list of publicly available datasets that can be used to learn person pose models. In addition, we test and evaluate 12 popular 2-stage models for 3D HPE with a monocular camera trained on public and custom datasets in unseen environments and scenes such as handball jump shots to assess the robustness and applicability of the methods in a new and unfamiliar sports domain and environment.

**Figure 1:**
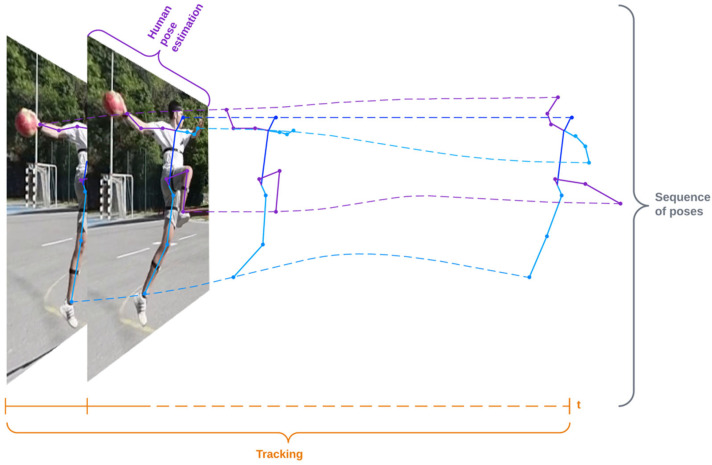
Creating a sequence of poses using human pose estimation to produce human skeleton keypoints and object tracking for grouping collected poses across frames (*t*) into a single sequence of poses.

Finally, to improve the performance of pose estimation methods for action recognition and comparison tasks where a sequence of aligned pose detection is a prerequisite, we have defined a method-independent pipeline that includes smoothing (to remove noise from the prediction) and retargeting (to standardize the distance between keypoints before pose estimation), and experimentally tested the effects on performance improvement on different models. The procedure for obtaining a sequence of poses is shown in Figure 1. Human pose estimation is used to create keypoints of the human skeleton, and object tracking is used to group poses collected through the sequence of frames into a series of poses corresponding to an activity.

The contributions of this work can be summarized as follows:

- Overview of the methods, models, and algorithms used in pose estimation and tracking with a monocular camera;
- Evaluation of 12 selected 2-stage pose estimation models based on deep learning in a 3D pose estimation task with a monocular camera trained on public and custom datasets to test the robustness of the model in the new sports domain and environment;
- Proposed method-independent pipeline for smoothing and retargeting 3D pose estimation sequences for action recognition and comparison tasks where an aligned pose sequence is a prerequisite;
- Evaluation of the prediction performance of 12 selected 2-stage deep learning models on a 3D pose estimation task when the proposed method-independent pipeline is used to smooth the estimated 3D sequences;
- Evaluation of selected 5 state-of-the-art tracking methods to assess the robustness of the models in an unseen sports environment.

The rest of the paper is organized as follows: Section 2 describes the methods for pose estimation using a monocular camera, as well as various approaches for improving the accuracy of pose estimation, methods for standardizing poses, and data sets for pose estimation. Section 3 describes tracking algorithms that allow detected poses to be linked in sequences in a multi-person environment along with tracking datasets, while Section 4 and Section 5 describe the evaluation of 3D pose estimation pipelines and tracking methods on public and custom datasets. The paper ends with a conclusion and discussion.

## 2. Pose Estimation

**Figure 2:**
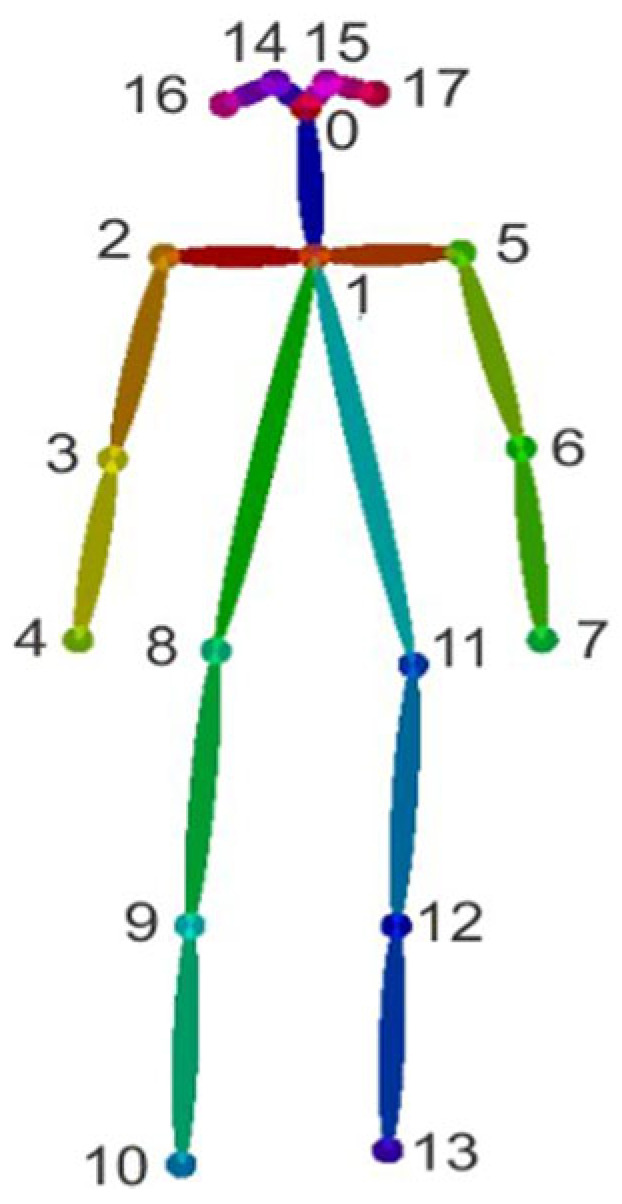
Standard 18-person keypoints in pose estimation.

Estimation of human posture is essentially a matter of identifying and classifying the joints of the human body so that a skeleton can represent the human body in such a way that each joint (arm, head, torso, etc.) important in representing a person’s posture is given as a set of coordinates and is connected to the adjacent keypoints. Typically, posture estimation is based on the determination of 18 standard keypoints representing important body parts and joints, as shown in Figure 2.

The goal of HPE is to design the representation of the human body in such a way that geometric information and information about the movement of the human body can be understood, further processed, and applied to specific tasks. At various stages of development, three different representations of the human body were considered: the skeleton-based model, the contour-based model, and the volume-based model. Today, however, the skeleton-based representation is predominant.

The evaluation of the human position can be done in the plane or in space, and 2D or 3D methods are therefore used to predict and represent the position of the human body.

Traditional approaches use 2D models to estimate the 2D position or spatial location of human body points from images and video frames and rely on hand-crafted low-level features such as Gaussian-oriented histograms (HOG), contours, colour histograms, and machine learning methods such as Random Forest to determine joints in the human body. However, all traditional methods have one problem: they only work when all body parts are visible and clearly represented. These problems are largely overcome by the use of deep neural networks, which can learn complex features and achieve higher accuracy when enough data is available. As a result, they are now predominantly used for all computer vision tasks, including human pose estimation.

Toshev and Szegedy [1] were the first to use a Deep Convolutional Neural Network (CNN) for the human pose estimation problem. They developed the DeepPose model, which yielded promising results and showed that the network can model poses with hidden and occluded joints. They also focused on further research on approaches based on Deep Learning.

**Figure 3:**
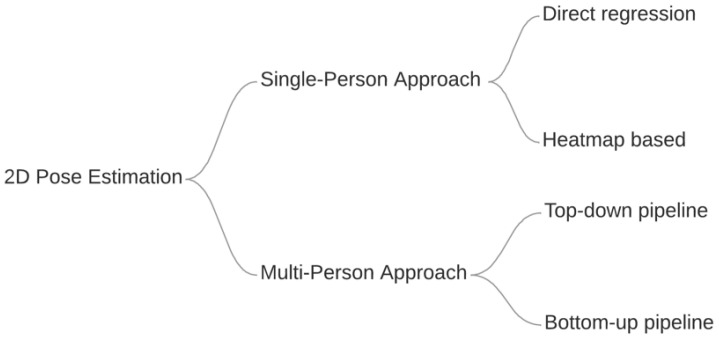
Taxonomy of pose estimation approaches based on Ref. [2].

Several Deep Learning based approaches have been introduced to achieve better pose estimation results, which according to Ref. [2] can be generally divided into two categories: Single Person Approaches and Multiple Person Approaches, as shown in Figure 3.

In the single-person approach, the pose of a person in an image is recognised based on the position of the person and an implicit number of keypoints, so it is essentially a regression problem. The multi-person approach, on the other hand, aims to solve an unconstrained problem, since the number and positions of the persons in the image are unknown.

### 2.1. The Single-Person Approach

The single-person approach is divided into two frameworks based on the keypoint prediction method: direct regression of keypoints from features (i.e., direct regression-based framework) or generating heatmaps and inferring keypoints via heatmap (i.e., heatmap-based framework).

#### 2.1.1. Direct Regression-Based Framework

Toshev and Szegedy presented DeepPose in Ref. [1], where they proposed a cascaded Deep Neural Network (DNN) regressor for predicting keypoints directly from feature maps. The model follows a simple architecture with convolutional layers followed by dense layers that generate (*x, y*) values for keypoints. Carreira et al. [3] proposed a method to iteratively refine the model output by feeding back error predictions, resulting in a significant increase in accuracy. Luvizon et al. [4] proposed a soft Argmax function to directly convert feature maps into common coordinates using a keypoint error distance-based loss function and a context-based structure to achieve competitive results compared to a heatmap-based framework. Sun et al. [5] proposed a structure-aware regression approach using a reparametrized pose representation with bones instead of joints. Bones are easier to recognize becausee they are more primitive and stable, cover a larger area, and are more robust to occlusion, making them easier to learn than joints. The presented results show an improvement in performance over previous direct regression based systems, but are also very competitive with heatmap based systems.

#### 2.1.2. Heatmap-Based Framework

**Figure 4:**
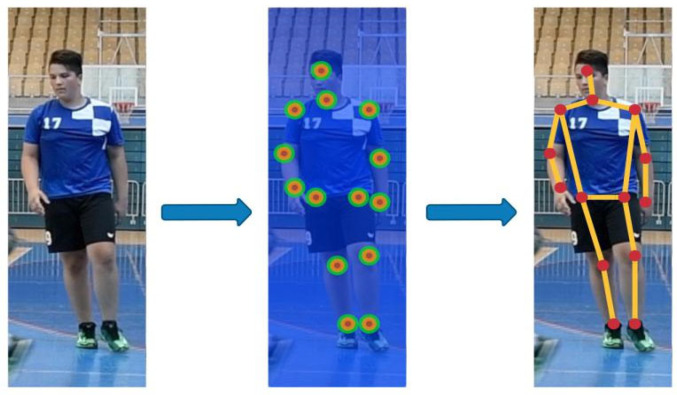
Heatmap poses estimation. It starts by creating heatmaps of all keypoints within the image, and then additional methods are used to construct the final stick figure.

Instead of predicting keypoints directly, an alternative approach can be used to create heat maps of all keypoints within the image. Then, additional methods are used to construct the final stick figure, as shown in Figure 4.

Chen and Yuille [6] proposed a graphical model with pairwise relations for adaptive use of local image measurements. Local image measurements can be used both to detect joints and to predict the relationships between joints. Newell et al. [7] designed a “stacked hourglass” network closely related to the encoder-decoder architecture, based on the sequential steps of pooling and upsampling before generating the final prediction set. They showed that repeated bottom-up and top-down processing with intermediary supervision is critical for improving performance in human pose detection. Later research commonly used a stacked hourglass network. Adversarial PoseNet [8] uses a discriminator to distinguish between real and fake poses, which are usually the result of a complex scene or occlusions. The discriminator learns the structure of the stick figure, and can thus decide whether a pose is real (reasonable as a body shape) or fake. The discriminator results are then used to further train the model for pose estimation. Chu et al. [9] use a multi-context attention mechanism that focuses on the global consistency of the entire human body and the description of different body parts. In addition, they introduce a novel Hourglass Residual Unit to increase the receptive field of the network. Martinez et al. [10] introduce a basis for 3D estimation of human poses that uses an hourglass network to predict 2D keypoints, which are then fed into a simple feed-forward network that provides a prediction of 3D keypoints.

### 2.2. The Multi-Person Approach

The multi-person approach is more complex because the number and positions of people in the image are not given. Therefore, the system must recognise keypoints and assemble an unknown number of people. Two pipelines have been proposed to deal with this task: a top-down pipeline and a bottom-up pipeline.

#### 2.2.1. Top-Down Pipeline

**Figure 5:**
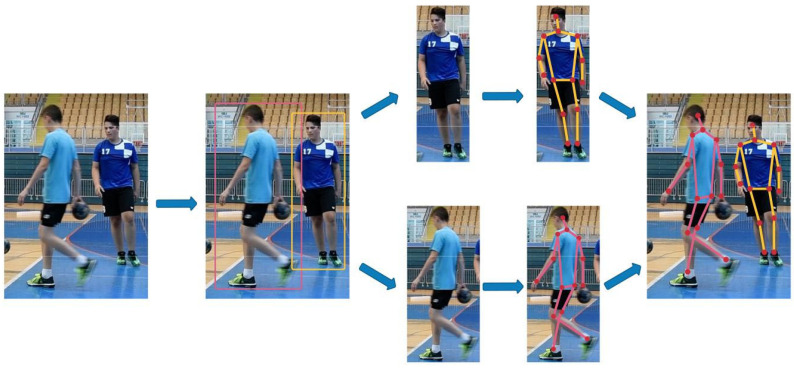
The top-down pipeline in multi-person approach for pose estimation. It starts by detecting all persons within an image and producing bounding boxes, on which a single-person approach is applied. The result are keypoints for each detected person, after which the pipeline may involve additional post-processing steps and improving the final results.

The top-down pipeline starts by detecting all persons within an image and creates bounding boxes around them. The next step is to use each of the detected bounding boxes and perform a single-person approach for each of them. The single-person approach creates keypoints for each detected person, after which the pipeline may include additional post-processing steps and enhancement of the final results, as described in Figure 5.

The top-down method was first proposed in the study by Toshev and Szegedy [1], where a face detector-based model was used to determine the bounding box of the human body. In the next step, a multilevel DNN-based cascade regressor was used to estimate the joint coordinates.

He et al. [11] developed a segmentation model as an extension of the Faster Region-Based Convolutional Neural Network (R-CNN) [12] model by adding a branch to predict object masks. The robustness and better results of the proposed model were improved by using a human pose estimation model. The Mask R-CNN simultaneously predicts the human bounding box and the human keypoints, which speeds up the recognition by sharing the features between the models.

Radosavovic et al. [13] used omni-supervised learning with the Mask R-CNN detector for challenging real-world data. Self-learning techniques were applied so that the predictions of the Mask R-CNN detector on unlabelled data were used as additional training data.

Fant et al. [14] used the sensitivity of a single-person pose estimation to bounding box detection. The authors developed a method to handle inaccurate bounding boxes and redundant detections by using a Symmetric Spatial Transformer Network (SSTN) and a Pose-Guided Proposals Generator (PGPG). Moreover, PGPG is used to greatly augment the training data by learning the conditional distribution of bounding box proposals for a given human pose. This adapts the single-pose estimator to handle human localization errors due to SSTN and parallel use of the single-pose estimator.

#### 2.2.2. Bottom-Up Pipeline

**Figure 6:**
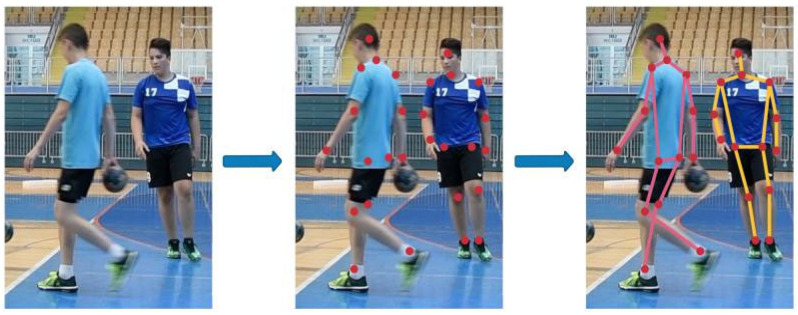
The bottom-up pipeline in multi-person approach for pose estimation. It starts by detecting all the keypoints in the image, which are then associated with human instances.

The bottom-up pipeline works like a reverse top-down pipeline and starts by detecting all keypoints in the image, which are then associated with human instances, as shown in Figure 6. Compared to the top-down pipeline, the bottom-up pipeline is likely to be faster because it does not detect human bounding boxes and does not perform pose estimation separately for each person detection.

The bottom-up multi-person pipeline for pose estimation was first proposed by Pishchulin et al. [15]. They formulated it as a joint problem of partitioning and labelling subsets. The model jointly determines the number of persons, their poses, spatial proximity, and occlusions at the part level. Their formulation implicitly performs non-maximum suppression on the set of keypoint candidates and groups them to form body part configurations that account for geometric and visual constraints. Insafutdinov et al. [16] improved the performance of the previously described method [14] in complex scenes by using a deeper neural network for better recognition of body parts and introducing new image-conditioned pairwise terms to achieve faster pose estimation.

Insafutdinov et al. made another improvement [17] by simplifying and reducing the body part relation graph, using current methods for faster inference, and shifting much of the inference about body part association to a feed-forward convolutional architecture.

Next, Cao et al. [18] proposed a non-parametric representation called Part Affinity Fields (PAFs) to learn the association of body parts to people in the image. Their model generates a set of confidence maps for body part positions and a set of vector fields of part affinities, which are finally parsed by greedy inference to output keypoints.

Newell et al. proposed associative embeddings [19], which is a method that simultaneously outputs detection and group assignment and outperforms bottom-up methods such as in Refs. [15,17,18], as well as a top-down method proposed in Ref. [14]. The embeddings serve as tags that encode a grouping: detection with similar tags should be grouped, i.e., body joints with similar tags should be grouped into one person.

Huang et al. [20] took a different direction in their search for performance benefits in a pose estimation task. They focused on the data processing problems arising from complex biased coordinate system transformations and keypoint format transformation methods. Therefore, they proposed Unbiased Data Processing (UDP), which consists of two techniques: an unbiased coordinate system transformation (achieved with elementary operations such as cropping, resizing, rotating, and flipping) and an unbiased keypoint format transformation (achieved by an improved keypoint format transformation between heat maps and keypoint coordinates).

A summary of the key differences between described methods is shown in Table 1.

### 2.3. 3D Pose Estimation

The 3D pose estimation aims to provide a complete and accurate 3D reconstruction of a person’s motion from a monocular camera or, more commonly, from 2D position keypoints.

Early studies focused on predicting 3D poses directly from images. Li and Chan first introduced the concept of predicting 3D poses using Deep Learning in Ref. [21] by constructing a convolutional neural network trained to regress 3D keypoints directly from the image. Their simple approach outperformed previous approaches that do not impose constraints on the definition of correlation between body parts. Tekin et al. [22] build on a similar idea to Ref. [21], but take advantage of using auto-encoders in latent space for 3D pose representation. First, they trained the auto-encoder to reconstruct a 3D pose given as input to the network and generated a pose representation in latent space (middle layer in the network). A CNN network was then trained to generate pose representations directly from images, rather than regressing keypoints directly, as was done in previous work. The resulting pose representation from the CNN network is then fed into the decoder network to generate a 3D pose. In addition, this approach enforces an auto-encoder that implicitly learns constraints about the human body, improving pose consistency and correlation between body parts without being explicitly trained. Pavlakos et al. [23] formulate 3D pose estimation as a 3D keypoint localization problem in a voxel space using a convolutional network to create keypoints heatmaps. The input to the network is a single image, and the output is a dense 3D volume with separate probabilities per voxel for each joint. To handle the high dimensionality and enable iterative processing, they incorporated a coarse-to-fine supervision scheme instead of using a single component with a single output.

Splitting the task of 3D pose estimation into two steps proved to be a better approach than directly predicting 3D poses from images and is more commonly used in recent studies. Martinez et al. [10] presented a simple deep feed-forward network that “lifts” 2D joint positions into 3D space, outperforming all previous methods. They analysed errors in previous approaches that predicted 3D keypoints directly from images and concluded that one of the main causes of errors stems from 2D pose estimation, which propagates errors further into later steps. By separating the two tasks, overall accuracy is improved because each step can be evaluated and improved separately.

Recent studies have focused primarily on improving estimation performance by evaluating temporal pose information across multiple images or frames. Hossain and Little [24] used the temporal information about a sequence of 2D joint position to estimate a sequence of 3D poses by using a sequence-to-sequence network of layer-normalized LSTM units. The proposed seq2seq network uses only the previous frames to understand the temporal context and produces predictions with errors uniformly distributed over the sequence. In Ref. [25], Pavllo et al. proposed a simple and effective approach for 3D human pose estimation based on dilated temporal convolution of 2D keypoint trajectories and a semi-supervised approach that exploits unlabelled video to improve performance when there is limited data. Their convolutional network achieves similar results to more complex LSTM sequence-to-sequence models and solves the problem of pose drift over long sequences of seq2seq models. In Ref. [26], Chen et al. solved the problem of missing information due to occlusions, out-of-frame targets, and inaccurate person detection by proposing a framework that integrates graph convolutional networks (GCNs) and temporal networks (TCNs). They proposed a human-bone GCN that models bone connections and a human-joint GCN based on a directed graph. By using the two GCNs, they can robustly estimate the spatial frame-wise 3D poses enough to work with occluded or missing information about human parts. In addition, a joint TCN was used to estimate the person-centred 3D poses across multiple frames and a velocity TCN was used to estimate the velocity of the 3D joints to ensure the consistency of the 3D pose estimation in successive frames. By using the two TCNs, 3D pose estimation can be performed without requiring camera parameters. Li et al. proposed a novel augmentation method [27] that is scalable to synthesize a large amount of training data for training 2D-to-3D networks, which can effectively reduce the bias of datasets. The proposed data evolution strategy extends an existing dataset through mutations and crosses of selected poses to synthesize novel human skeletons to expand the dataset in the order of 10^7^. In addition, they proposed a novel 2D-to-3D network that contains a cascaded 3D coordinate regression model and where each cascade is a feed-forward neural network.

A summary of the key differences between described methods is shown in Table 2.

### 2.4. Occlusion

Occlusion is the predominant problem in estimating human posture, and a number of papers have attempted to solve this problem. Iqbal and Gall [28] considered multiple person pose estimation as an association problem between two persons, and used linear programming to solve the association problem anew for each person. Chen et al. proposed a novel network structure called Cascaded Pyramid Network (CPN) [29], which includes GlobalNet and RefineNet. The GlobalNet is used to locate visible keypoints, while the RefineNet is used to handle keypoints that are difficult to see or hidden. Fang et al. [14] used Non-Maximum Suppression to solve the occlusion problem and eliminate redundant poses, the problem caused by redundant detections. A similar approach was implemented in Ref. [30] to eliminate redundant detections.

### 2.5. Metrics

In the early works, frequently used metric was the Percentage of Correctly estimated body Parts (PCP) [31]. In PCP, a limb is considered to be detected and to be a correct part if the distance between the predicted and true joint position is less than the bone length multiplied by a chosen factor. The true joint position of the limb is at most half the length (PCP at 0.5), as shown in Equation (1). Another widely used metric is PCK (Percentage of Correct Keypoints) [32] and its variant PCKh, shown in Equation (2). In both metrics, a joint is considered detected and correct if it is within a certain number of pixels from the ground truth joint, determined by the height and width of the person bounding box (or person’s head in the case of PCKh). More recent metrics are Percentage of Detected Joints (PDJ) [1], shown in Equation (3), and Object Keypoint Similarity (OKS) [33], shown in Equation (4). PDJ considers a joint to be correctly detected if the distance between the predicted joint and the true joint is within a certain fraction of the diagonal of the bounding box. OKS is calculated from the distance between the predicted points and the ground truth points normalized by the person’s scale. The OKS metrics show how close the predicted keypoint is to the ground truth, with a value from 0 to 1. The final performance calculation usually involves thresholding the OKS metrics and calculating the Average Precision (AP) and Average Recall (AR), as shown in Equation (5). Mean Per Joint Position Error (MPJPE) is the most commonly used metric. MPJPE, Equation (6), calculates the Euclidean distance between the estimated 3D joint and the ground truth position, and the final score is calculated by averaging the distances across all frames. A common addition in the evaluation process is to align the poses before calculating the metrics. The most widely used alignment method is Procrustes alignment, which relies on Procrustes analysis to compare the two poses and align them on all axes. Metrics that use Procrustes alignment are usually marked with the prefix PA (e.g., PA-PCK, PA-MPJPE).

PCP, PCKh, and PDJ metrics are calculated as follows:

(1) $$PCP=\frac{\sum_{i=0}^{n}bool(d_{i}<0.5\times limb\_length_{i})}{n}$$

(2) $$PCKh=\frac{\sum_{i=0}^{n}bool(d_{i}<0.5\times height\_of\_the\_head)}{n}$$

(3) $$PDJ=\frac{\sum_{i=1}^{n}bool(d_{i}<0.05\times diagonal)}{n}$$

where $d_{i}$ is the Euclidean distance between the ground truth keypoint and predicted keypoint, bool(condition) is a function that returns 1 if the condition is true and 0 if it is false, n is the number of keypoints on the image. limb_length, head_height, and diagonal are expressed as the number of pixels per the model predictions expressed in pixels as well.

In PDJ, the diagonal is calculated from the bounding box using the Pythagorean theorem, i.e., $diagonal=\sqrt{\left(height^{2}+width^{2}\right)}$.

The OKS metric is calculated as follows:

(4) $$OKS=exp\left(-\frac{d_{i}^{2}}{2s^{2}k_{i}^{2}}\right)$$

where $d_{i}$ is the Euclidean distance between the ground truth keypoint and predicted keypoint, s is the square root of the object segment area (scale), and k is a per-keypoint constant that controls fall off.

AP and AR metrics with Precision and Recall formulas are calculated as follows:

(5) $$\begin{array}{r} Precision=\frac{TP}{TP+FP}\\ Recall=\frac{TP}{TP+FN}\\ AP=\overset{n}{\underset{i=0}{\sum }}Precision_{i}\\ AR=\overset{n}{\underset{i=0}{\sum }}Recall_{i} \end{array}$$

where n is the number of keypoints, *TP* represents True Positives, *FP* represents False Positives, and *FN* represents False Negatives.

The MPJPE metric is calculated as follows:

(6) $$E_{MPJPE}\left(f,\varphi \right)=\frac{1}{N_{\varphi }}\sum_{i=1}^{N_{\varphi }}\|P_{f,\;\varphi }^{\left(f\right)}\left(i\right)-P_{gt,\;\varphi }^{\left(f\right)}{\left(i\right)\|}_{2}$$

where f denotes a frame and φ denotes the corresponding skeleton. $P_{f,\;\varphi }^{\left(f\right)}\left(i\right)$ is the estimated position of joint i and $P_{gt,\;\varphi }^{\left(f\right)}\left(i\right)$ is the corresponding ground truth position. $N_{\varphi }$ represents the number of joints.

### 2.6. Standardization—Spatial Alignment, Normalization, and Retargeting

Because images may be of different sizes, a person may appear in a different part of the image, requiring a preprocessing step to allow consistent calculation of accuracy metrics. In addition, the use of accelerometers or motion capture sensors such as mocaps complicates the task of accurately evaluating pose estimation methods. In these cases, it is suggested to apply pose transformations to remove potential errors caused by inappropriate preprocessing.

A simple solution is to normalize the resulting keypoint coordinates by treating them as an L2-normalized vector array. In addition, the poses can be aligned by a selected pose point (e.g., a point between the hips [34]) or by Procrustes analysis, as in Refs. [35,36,37,38]. In our experiments, we defined and applied a simple normalization procedure where the person is scaled so that the height of the person is 1, and we will here refer to it as the h-norm. The H-norm assumes that there is at least one frame in the sequence in which the person is stretched, finds that frame, and then scales the person based on this frame. The height of the person is calculated as the distance between the nose and the foot keypoints, taking into account the foot that is further away from the nose. Finally, the h-norm sets the height of the person in the selected “stretched” image to 1 and scales the other images accordingly.

A more advanced solution is to use a retargeting method, as proposed in Refs. [39,40,41,42], which transfers the joint angles from the predicted pose to a standardized skeleton. The result is a new pose where the limbs are always the same length, which also solves the problem of pose estimation models with small variations in keypoint detection. For example, a hand may be detected at the wrist or in the palm region, and this mismatch of detections results in an incorrect limb length.

**Figure 7:**
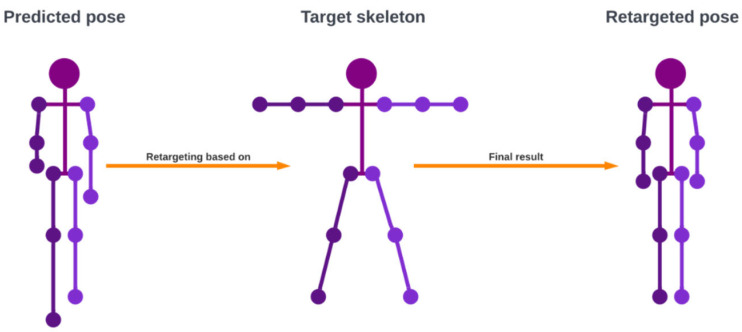
An example of pose retargeting where the predicted pose is retargeted based on the target skeleton. Retargeting will translate the joint angles from the predicted pose to a standardized skeleton, thus ensuring that a pose has the same lengths of limbs.

In this experiment, we applied the simplest way of implementing a retargeting method, which is to use a direction vector. A keypoint $P_{t}$ is retargeted using the root keypoint $P_{r}$ by subtracting the two points to produce a direction vector ${\vec{p}}_{t}$ (the magnitude vector). Then, we rescale ${\vec{p}}_{t}$ to the distance between the targeted root point $T_{r}$ and the targeted keypoint $T_{t}$ to produce the direction vector ${\vec{t}}_{t}$. Finally, we add ${\vec{t}}_{t}$ to point $T_{r}$ producing the retargeted keypoint $T_{t}^{\prime }$. An example of the retargeted pose is shown in Figure 7.

### 2.7. Datasets

Several publicly available datasets are provided for various image processing tasks and domains. Among the most popular datasets are COCO [43] and ImageNet [44], which contain many tagged images of various objects in the real-world conditions.

It is necessary to properly collect the data and prepare it for machine learning for various tasks such as image classification, object detection, object localization, object segmentation, object tracking, etc. For image classification, the images are annotated with a label corresponding to the class of the object that exists on the scene; for detection, the objects in the scene are surrounded by a bounding box, or the image area corresponding to the object is segmented. Finally, the skeleton of the object should be labelled for pose estimation.

**Figure 8:**
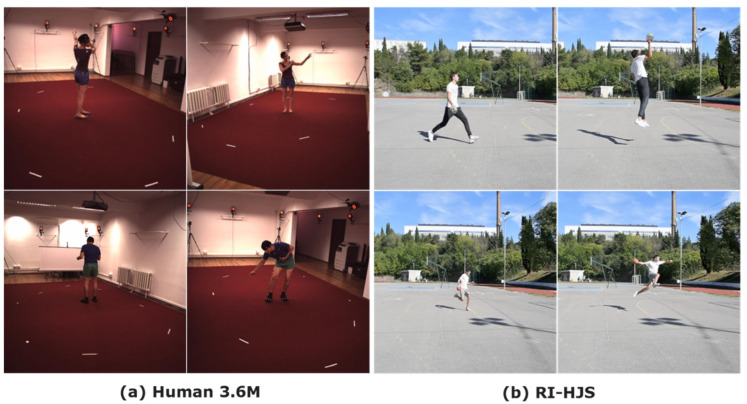
Examples from the Human3.6 dataset (**a**) and the RI-HJS dataset (**b**) for 3D pose estimation.

The most well-known dataset in the field of pose estimation is the Human3.6M dataset [45]. It consists of 3.6 million human poses and corresponding images captured with a motion capture system. The dataset contains 11 actors performing 17 activities (discussing, smoking, taking pictures, talking on the phone, etc.). Examples from the dataset are shown in Figure 8a.

There are also appropriate datasets with images or video sequences that are specific to a particular domain. For example, in the sports domain, data on Olympic sports are very popular for classifying sports scenes and are collected in the Olympic Sports Dataset [46], and SVW [47] contains short sequences of actions related to 16 and 30 sports, respectively. There are also specialized databases of videos related to specific sports, such as UNIRI-HBD [48], for researching the performance of athletes in handball, basketball [49], and volleyball [50].

Older publicly available image datasets such as KTH [51] and Weizmann [52] were filmed under controlled conditions with fewer actors, while on the other hand, datasets such as HACS [53] and Kinetics 700-2020 [54] were filmed under real-world conditions and contain many more classes and data. Kinetics, for example, is a large dataset (with 400 to 700 classes corresponding to different human activities depending on the version) that contains manually tagged videos downloaded from YouTube. Other popular datasets in the sports domain are UCF Sports Action Data Set [55] and Sports-1M [56].

In the experimental part of this work, we prepared and used our own dataset of handball scenes collected in Rijeka (RI-HJS). Handball is an Olympic team sport played with a ball and is very popular in Europe, but is not represented in the aforementioned databases for training models for sports scenes. RI-HJS contains 21 short clips with an average length of 9 s, in which 2 different players perform several handball jump shots. Both players were equipped with Wear-Notch motion capture sensors to capture the ground truth positions of the joints. The documentation states that the static accuracy of the Wear-Notch sensors is approximately 1–2° yaw/tilt/roll. We used a single still camera with 1920 × 1080 resolution positioned on the tripod 1.5 m from the ground, while the players were about 7–10 m away from the camera. Examples from the dataset are shown in Figure 8b.

## 3. Tracking

Multiple object tracking (MOT) in videos is an actively researched area in computer vision, and in this paper we present the main methods to achieve the best performance. The main goal of multiple object tracking (MOT) is to track the position and identity of multiple objects so that each object is assigned the same unique ID in each frame in which it appears in the video.

Tracking produces the best results when objects are moving uniformly, in the same direction, and without occlusions. Examples where tracking works well include runners chasing each other on the edge of a playground, or cars moving in the same direction and at the same speed on the road without being obscured by objects. However, this is usually not a realistic scenario, especially in team sports where many players change the direction of movement, speed, distance from the camera, position, and activity performed. They also frequently enter and exit the camera’s field of view, so they are visible in some shots and not in others. They also stand very close to each other to interfere with the opponent and prevent him from taking appropriate action. They often occlude each other, and because of the obscuring, it is difficult to detect their whole body. The players of the team wear the same jerseys, so they can be identified only by the number on the jersey or some details such as hair colour or sneakers. In dynamic scenes, tracking more objects is still a big challenge. However, thanks to the improved performance of object and skeleton detectors, even in crowded scenes, and improved computer performance, tracking by detection has become the leading paradigm for MOT.

In tracking by detection, the tracking algorithm relies on the results of the object detectors in each frame and combines the information

**Figure 9:**
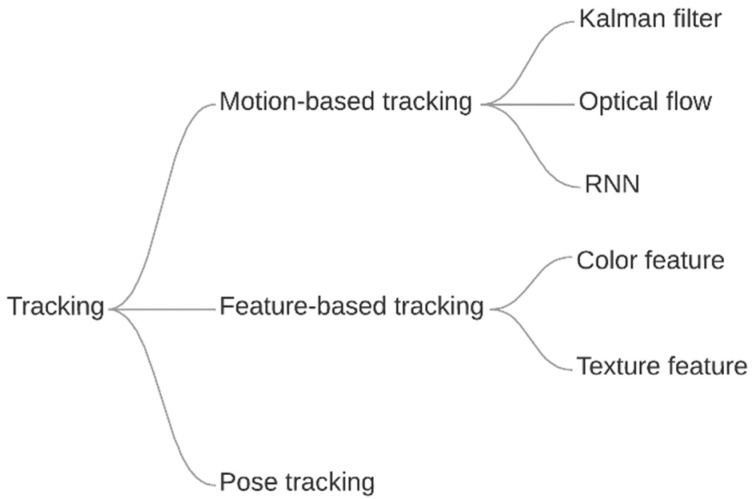
Taxonomy of the tracking methods.

**Figure 10:**
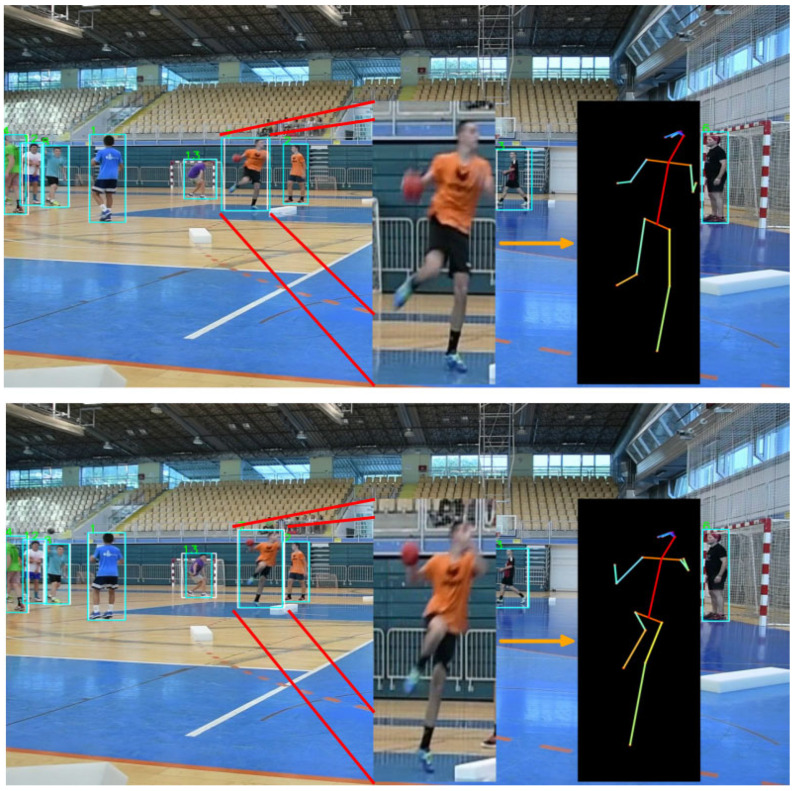
Two frames of a tracked player executing a jump shop where poses are estimated and performed necessary transformation. Blue bounding boxes visualize the detectors’ outputs, while white bounding boxes visualize the tracking algorithm bounding box prediction. To standardize pose sizes because players can be further away or closer to the camera, we perform transformations to the pose (i.e., standardization).

In general, multiple object tracking is about detecting bounding boxes of an object in successive frames and a method to map them between image sequences, thus creating object trajectories. The taxonomy of tracking methods described in this paper is shown in Figure 9, while an example of tracking on an image is shown in Figure 10.

### 3.1. Motion-Based Tracking

Motion-based detection methods mainly consist of background subtraction and difference between adjacent frames. Motion models are often robust and computationally light, but their performance is heavily affected by noise and depends heavily on frame registration, so even small errors in frame registration or illumination changes can lead to large errors in motion-based object detection. A typical traditional approach has been background modelling using Gaussian Mixtures (GMM). However, since these are not capable of detecting objects in the scene, several methods have been proposed that combine background distribution estimation with numerous filters for video post-processing and object detection.

Motion-based tracking involves recording the motion of an object in a source video clip, then analysing its motion and trajectory, and using this motion behaviour to predict a target object in a sequence of video clips. A well-known example is the use of the Kalman filter [57,58] to estimate the position of a linear system, assuming that the errors are Gaussian. The Kalman filter [59] is an algorithm that uses a series of measurements observed over time, including noise and other inaccuracies. It provides estimates of target variables that are usually more accurate than estimates based on a single measurement. The Kalman filter is usually combined with various techniques to represent object features or to improve the estimate of the target position [60,61,62,63]. One of the most popular tracking systems that use the Kalman filter is Simple Online and Realtime Tracking (SORT) [63], a system based on state estimation techniques designed for online tracking where only previous and current frames are available. SORT uses the Kalman filter to predict object position in the current frame based on the previous frames, i.e., object movement across previous frames, along with the Hungarian matching algorithm [64] to perform data mapping and assignment on the same track (connecting bounding boxes across frames). The Hungarian algorithm searches for the optimal bounding box that best matches a given bounding box in the previous frame, given a cost allocation function that depends only on the parameters of the bounding box. The parameters used to assign objects on the track are the Euclidean distance of each detected object from the predicted centre of the last object on the track and the difference in bounding box size between the detected object and the last assigned object on the same track. This algorithm does not consider visual features and similarities between objects in successive frames. An object is assigned to a track if the reliability of the detector is higher than the set threshold. If the number of detected objects exceeds the number of currently active tracks, new tracks are created and initialized with the new object.

In some works [65,66,67,68,69,70], optical flow was used for object tracking by separating the moving foreground objects from the background and generating an optical flow field vector for the moving object. Optical flow is a low-level feature determined from the time-varying image intensity between subsequent frames. The moving point in the image plane estimated from successive video frames, e.g., by using the Lucas–Canada method [71], generates a 2D path *x*(*t*) ≡ (*x*(*t*), *y*(*t*))**^T^** with coordinates at the centre of the camera and the current direction of motion described by the velocity vector d*x*(*t*)/d*t*. The 2D velocities of all visible points in the image form a 2D vector field of motion, where the magnitude corresponds to the velocity of motion and the angle represents the direction of motion.

Other works, such as Refs. [72,73,74,75], use Recurrent Neural Network (RNN) to learn the motion behaviour of objects and use them for object tracking, usually applying them to bounding box coordinates. RNNs have connections that feed activations from an input in a previous time step back into the network, called memory cell units, which affect the output for the current input. These activations from the previous time step can be held in the internal state of the network to model long-range dependencies, so that the temporal context of the network is not limited to a fixed window and the network can model sequences such as video images in action recognition.

### 3.2. Feature-Based Tracking

Feature-based tracking is a method in which objects (features) in the data are first segmented, and then these segmented objects are tracked (correlated) in successive time steps based on the representation of their appearance, i.e., colour, texture, shape, and so on. Wojke et al. [76] improved the method proposed in Ref. [63] SORT by introducing a deep association metric. This is achieved by capturing object features within the bounding box to enable object tracking through longer occlusion periods, thus reducing the number of identity switches. Subsequent work, such as Refs. [77,78,79], has focused on improving object associations between frames using different methods or constructing a single model to perform object tracking and association. Further improvements were made by segmenting objects within the detected bounding box to eliminate unnecessary information (background, other objects, etc.), as proposed in Ref. [80], and subsequent improvements to the new approach [81,82,83].

### 3.3. Pose Tracking

Iqbal et al. [84] first formulated the problem of pose estimation and tracking for multiple persons and presented a sophisticated “Multi-Person PoseTrack” dataset. The authors proposed a method to solve this problem by representing the joint body detection with a spatiotemporal graph and solving an integer linear program to partition the graph into subgraphs corresponding to the plausible body pose trajectories for each person. Xiu et al. proposed a PoseFlow method [85], which consists of two techniques, namely, Pose Flow Builder (PF-builder) and Pose Flow non-maximum suppression (PF-NMS). PF-Builder is used to associate the cross-frame poses pointing to the same person by iteratively constructing a pose flow using a sliding window, where PF-NMS uses the pose flow as a single unit in NMS processing to stabilize tracking. Doering et al. [86] proposed a temporal model that predicts temporal flow fields, i.e., vector fields that indicate the direction in which each body joint will move between two successive frames. Raaj et al. [87] built on the Part Affinity Fields (PAF) [18] representation and proposed an architecture that can encode and predict Spatio-Temporal Affinity Fields (STAF). Their model encodes changes in the position and orientation of keypoints over time in a recurrent manner, i.e., the network takes STAF heatmaps from previous frames and estimates them for the current frame. Bao et al. [88] proposed a framework for pose-aware tracking-by-detection that combines pose information with methods for detecting people in videos and associating people. The system uses prediction of the location of people in the detection phase, and thus uses temporal information to fill in the missing detections. In addition, the authors propose a Pose-guided Graph Convolutional Network (PoseGCN) for person association, a modelling task that uses the structural relationships between person and the global features of a person.

**Figure 11:**
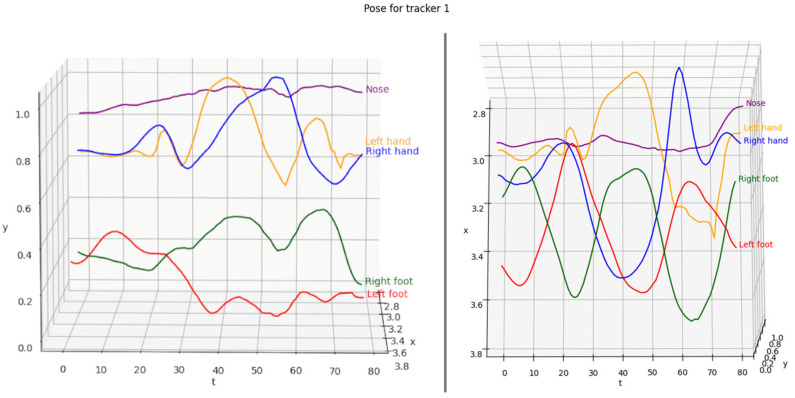
A 3D plot visualization of the 2D sequence joints in space and time when executing a jump shot, showing a side and top view of the plot.

In Ref. [89], Bazarevsky et al. focused on developing a lightweight method for estimating and tracking the single-person pose. They followed the top-down pipeline and used a face detector and certain computations to determine the width and height of a person’s bounding box, which made the detection fast. For the pose estimation step, the authors chose a combined heatmap, offset, and regression approach, using heatmaps and offset losses only during training. Kong et al. [90] proposed a framework consisting of the Pose-based Triple Stream Network (PTSN) and an online multi-state matching algorithm. PTSN is responsible for computing the similarity values between the historical tracklets and the candidate detection in the current frame. The values come from three network streams that model three pose cues, i.e., pose-based appearance, movements, and athlete interactions. An example of a tracked 2D pose sequence over 80 frames is shown in Figure 11.

### 3.4. Metrics

The evaluation of tracking algorithms usually involves a number of metrics. The most basic metric is the number of ID switches (IDsw) [91], which counts how many times an algorithm has switched (or lost) an object ID, as shown in Equation (7). An improvement on the IDsw metric is the IDF1 metric [92], which is computed as the ratio of correctly identified detections to the average number of detections based on ground truth and computed detections. ID precision and ID recall provide information about tracking tradeoffs. At the same time, the IDF1 score allows all trackers to be ranked on a single scale that balances identification precision and recall by their harmonic mean (see Equation (8)).

The most widely used metric is Multiple Object Tracking Accuracy (MOTA) [93], which combines three sources of error: false positives, missed targets, and identity switches into a single number, as shown in Equation (9). Another popular metric is Multiple Object Tracking Precision (MOTP) [93], which calculates the offset between the annotated and predicted bounding boxes, as shown in Equation (10). Finally, the Mostly Tracked targets (MT) [94] metric measures tracking completeness by calculating the ratio of trajectories covered by a track hypothesis to at least 80% of their respective lifetimes. The metric ML (Mostly Lost Targets [94]) is a complement to MT, which computes the ratio of trajectories covered by a track hypothesis during at most 20% of their respective lifetimes.

The IDsw metric is calculated as follows:

(7) $$IDsw_{t}=\overset{n}{\underset{i=0}{\sum }}bool(ID{\left(o_{i}\right)}_{t-1}\neq ID\left(o_{i}{)}_{t}\right)$$

where t is the frame index, n is a number of objects in the frame, o is the tracked object, and bool(condition) is a function that returns 1 if the condition is true and 0 if it is false.

The IDF1 metric is calculated as follows:

(8) $$IDF_{1}=\frac{2\;IDTP}{2\;IDTP+IDFP+IDFN}$$

where IDTP represents the number of correctly identified objects, IDFP represents the number of falsely identified objects, while IDFN represents the number of objects detections that fall outside the valid region of its corresponding ground truth.

The MOTA metric is calculated as follows:

(9) $$MOTA=1-\frac{\sum_{t}FN_{t}+FP_{t}+IDSW_{t}}{\sum_{t}GT_{t}}$$

where t is the frame index, and GT is the number of ground truth objects.

The MOTP metric is calculated as follows:

(10) $$MOTP=\frac{\sum_{t,i}d_{t,i}}{\sum_{t}c_{t}}$$

where $c_{t}$ denotes the number of matches in frame t and $d_{t,i}$ is the bounding box overlap of target i with its assigned ground truth object.

### 3.5. Datasets

Currently, the most popular and widely used dataset is Multiple Online Tracking (MOT) [95], which contains seven different indoor and outdoor scenes of public places with pedestrians as the objects of interest. The dataset provides detections of objects in the video frames using three detectors: SDP, Faster-RCNN, and DPM

Datasets where most people have similar appearance, such as sports and dance datasets, can greatly affect methods that rely on appearance as a tracking feature, and it is important to evaluate models in such scenarios. This type of dataset includes DanceTrack [96] and SportsMOT [97]. DanceTrack consists of 100 videos with a total of more than 10^5^ annotated frames and contains dancers in static scenes with uniform appearance, various movements, and frequent transitions. SportsMOT consists of 240 videos with a total of more than 10^5^ annotated frames and contain 3 types of scenarios: Basketball, Football, and Volleyball, covering outdoor and indoor scenes as well as different camera angles.

Datasets such as TAO [98] and GMOT [99] aim to evaluate the generality of tracking models and encourage tracking methods to generalize to different scenarios and objects, rather than overfitting to a single scenario and benchmark. TAO contains 2907 videos taken in different environments where multiple object categories are annotated (e.g., car, truck, animal, etc.), and is currently the most diverse tracking dataset with 833 different object categories annotated for tracking. GMOT contains 40 videos of complex scenes evenly distributed across 10 object categories. Some of the main features of the dataset are: over 80 objects of the same class can appear in 1 frame and annotations are done manually with careful inspection in each frame, occlusion, target entry or exit, motion, deformation, etc.

**Figure 12:**
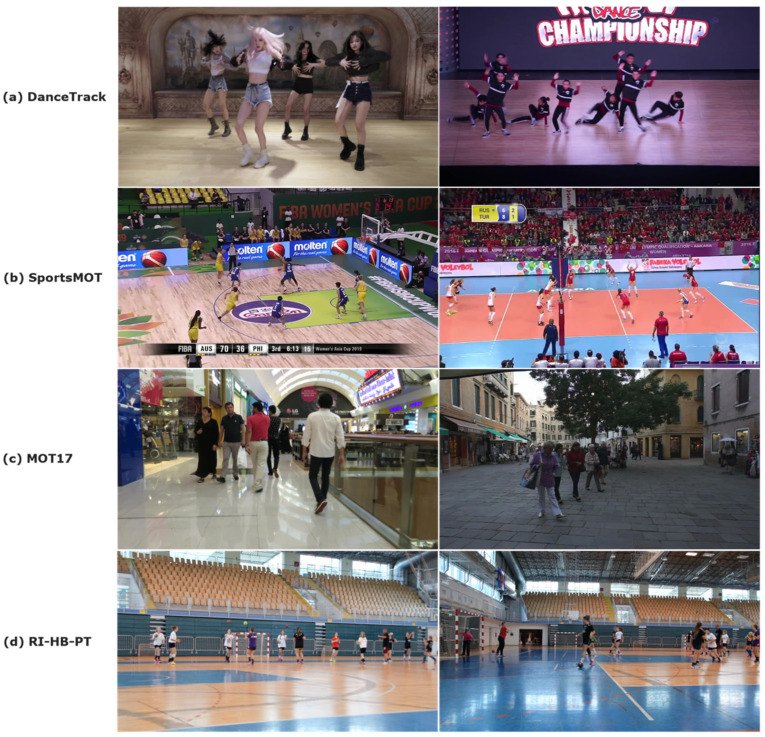
Examples from the tracking datasets DanceTrack (**a**), SportsMOT (**b**), MOT17 (**c**), and RI-HB-PT (**d**).

In this paper, for model testing purposes we use our custom dataset of 20 players practicing different handball actions during training sessions in Rijeka (RI-HB-PT) to test the pose estimation models. RI-HB-PT contains 2 videos with a total of 22,676 frames and 216,601 bounding box annotations. The training is very dynamic, and there is a lot of occlusion as players pass each other very often. We used a single still camera (1920 × 1080) positioned on the tripod 1.5 m from the ground, while the player was about 5–10 m away. Examples from some data sets are shown in Figure 12.

## 4. Evaluation of the 3D Pose Estimation Methods

We selected some well-known and well-performing methods for 2D and 3D pose estimation to evaluate on the Human3.6M dataset [45] and on our own RI-HJS dataset of handball players performing a jump shot.

The Human3.6M dataset was selected because it is considered the benchmark dataset in the field of pose estimation and contains 3.6 million human poses commonly used for training pose estimation models.

RI-HJS is a customised dataset of handball scenes. We used this dataset to evaluate the robustness of models learned on a large number of standard poses from Human3.6M, and to estimate the level of generality that can be achieved on new examples from handball for which they have not been trained. Important for testing the models is the fact that the handball examples used are from the new domain, but have similar indoor conditions as other indoor sports or ordinary activities, with artificial lighting, with the player moving quickly on the field and performing different techniques and actions with the ball.

The goal of this experiment is to find a combination of models that provides the best overall results in an unseen sports environment. We considered 2D pose estimation methods: PoseRegression (https://github.com/dluvizon/pose-regression, accessed on 1 February 2022) [4], ArtTrack (https://github.com/eldar/pose-tensorflow, accessed on 1 February 2022) [17], Mask R-CNN (https://github.com/facebookresearch/detectron2, accessed on 1 February 2022) [11], and UDP-Pose (https://github.com/HuangJunJie2017/UDP-Pose, accessed on 1 February 2022) [20], and 3D pose estimation models: GnTCN (https://github.com/3dpose/GnTCN, accessed on 1 March 2022) [26], EvoSkeleton (https://github.com/Nicholasli1995/EvoSkeleton, accessed on 1 March 2022) [27], and VideoPose3D (https://github.com/facebookresearch/VideoPose3D, accessed on 1 March 2022) [25]. All 3D pose estimation models make predictions based on the results of 2D estimation models. Thus, there are 12 possible combinations of models for a final 3D prediction from an image. The 2D models PoseRegression and ArtTrack were trained using the MPII [100] training dataset, while the Mask R-CNN and UDP-Pose models were trained using the COCO 2017 training dataset.

In addition, all three 3D models, i.e., GnTCN, EvoSkeleton, and VideoPose3D, were trained with the Human3.6 training dataset, which allows for fair evaluation and comparison. Further details of the training are given in Table 3. Model combinations were evaluated using the Human3.6M validation dataset and our custom dataset of handball players executing a jump shot collected in Rijeka (RI-HJS), as described in Section 2.6. Experiments that used top-down methods were given ground truth bounding boxes to eliminate object detector errors, i.e., to evaluate only the accuracy of the pose estimation methods.

The final results are shown in Table 4 with respect to the metrics PA-PCK and PA-MPJPE described in Section 2.5. The best results for PA-PCK is when it scores 100%, and for PA-MPJPE when is 0. The KSM and KSM + RET columns in Table 4 show the improvement in the performance of the metrics when the proposed Kalman smoothing is applied to the predicted sequence and pose retargeting. KSM means Kalman smoothing is applied to the predicted sequence to remove the noise and oscillations of the keypoints generated by the HPE method. Kalman is applied separately to all axes of the coordinate system (XYZ) for each keypoint to independently smooth the time series of keypoints across the sequence. KSM + RET means that a Kalman filter is applied to the predicted sequence for smoothing, while retargeting is applied to both the predicted and the ground truth sequence.

### 4.1. Discussion of the Pose Estimation Results

**Figure 13:**
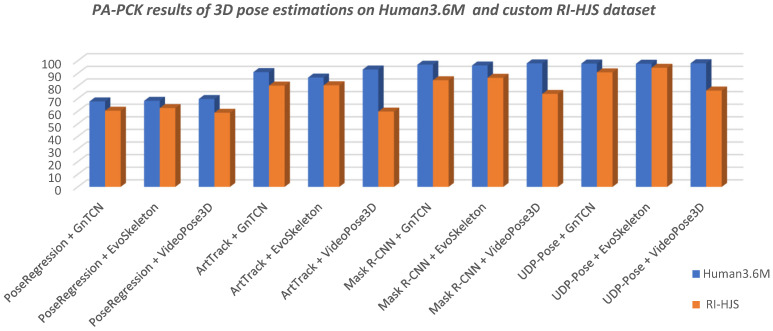
Comparison of the 3D pose estimation model results in terms of PA-PCK on Human 3.6M and custom RI-HJS datasets (higher is better). All models experienced a significant drop in performance on the RI-HJS dataset, except the two-step model UDP-Pose + EvoSkeleton, which retained high accuracy, showing robustness in an unseen environment. It is interesting to note that all two-step models that use VideoPose3D experienced the largest performance drop compared to other models.

**Figure 14:**
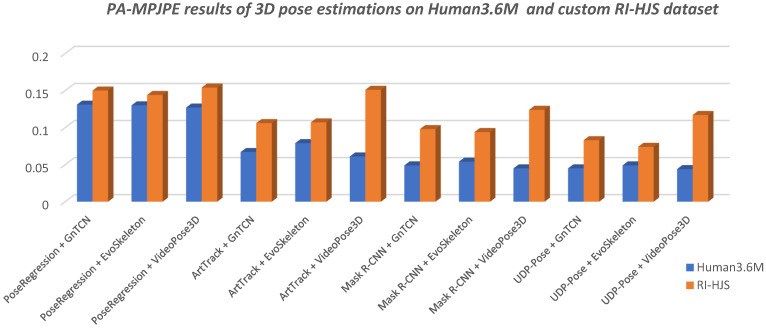
Comparison of the 3D pose estimation model results in terms of PA-MPJPE on Human 3.6M and custom RI-HJS datasets (lower is better). The comparison shows a significant drop in performance on the RI-HJS dataset, which is not surprising given that the models have never seen uncommon poses such as the handball jump-shot from the RI-HJS dataset. Two-step models that use VideoPose3D are more prone to errors due to unseen data, as they have the largest performance drop.

Table 4 shows that the tested models scored much better on the Human3.6M dataset than in the custom RI-HJS datasets in PA-PCK and PA-MPJPE metrics (Figure 13 and Figure 14). Better results on the Human3.6M dataset than on the custom dataset have been expected, given that all 3D models were pretrained on the training set of the Human3.6M dataset.

**Figure 15:**
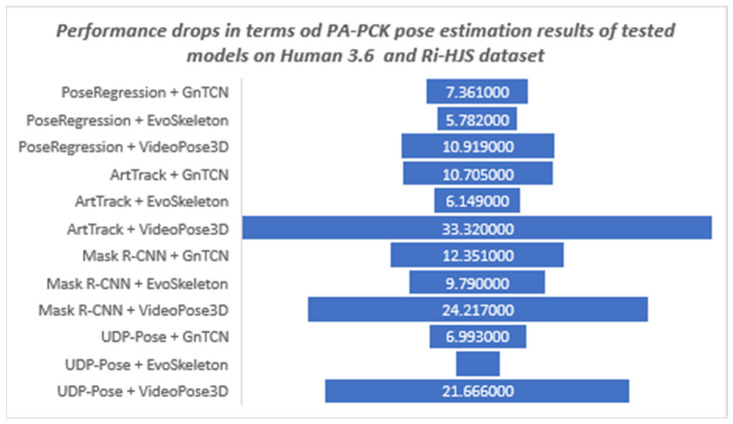
The robustness of the tested 3D models trained on the Human3.6M dataset shown as a difference of obtained results and performance drops between PA-PCK pose estimation results on Human 3.6M and custom RI-HJS datasets.

The lower performance values for the user-defined dataset indicate that the tested models are not robust enough to be applied to new environments without retraining. Figure 15 shows the differences between the results on the Human 3.6M test set and the results on the user-defined set obtained by the models trained on the Human 3.6M training set. It is interesting to note that the difference in the performance drop ranges from 3% to more than 33%. It should be noted that the UDP-Pose + EvoSkeleton model achieved almost the same high level of performance in the new custom set. In other words, all the tested models had the lowest performance drop when combined with the EvoSkeleton model, which ranged from 3% to a maximum of 10%, suggesting its robustness and its ability to be used in new sports scenes such as the handball scenes tested. The videoPose3D model, on the other hand, had the largest drop in performance regardless of which model it was used with; more specifically, it had a significant drop in performance of over 20% with all models except PoseRegression, where the drop was also significant but only half as large (about 11%).

Overall, the models using UDP-Pose for 2D pose estimation were found to perform better, which is not surprising since the authors reported better results than Mask R-CNN.

**Figure 16:**
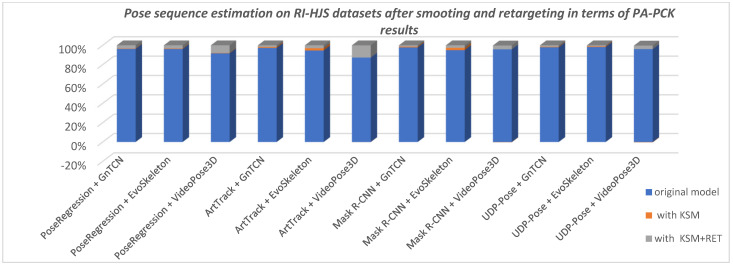
Comparison of pose sequence estimation in terms of PA-PCK on custom RI-HJS datasets (higher score is better). Two-step models that use EvoSkeleton show a significant improvement when using smoothing on the sequence of poses, showing the lack of consistency in the process of “lifting” 2D keypoints to 3D space. When using retargeting on the ground truth and smoothed predicted sequence, the results are significantly improved, indicating that all models lack an understanding of the human skeleton structure, which is especially true in the case of VideoPose3D.

**Figure 17:**
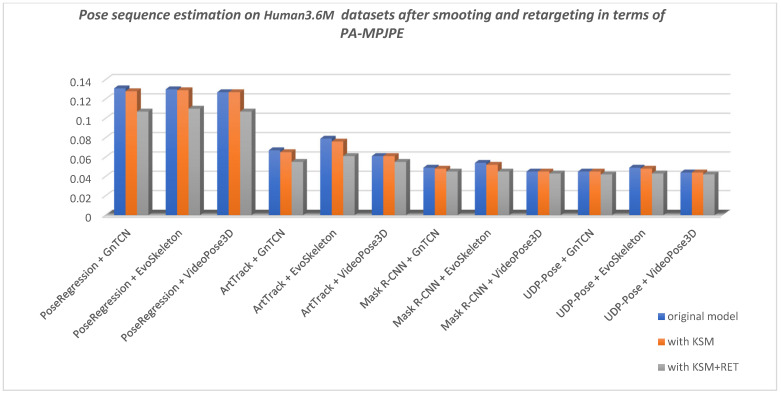
Comparison of pose sequence estimation in terms of PA-MPJPE measure on Human3.6M datasets (lower is better). All models show a slight improvement when using smoothing on the sequence of poses, showing the lack of consistency in the detection location of keypoints and “lifting” 2D keypoints to the 3D space. An exception to this conclusion is the VideoPose3D model, which constructed a smooth sequence of poses by utilizing temporal information. When using retargeting on the ground truth and smoothed predicted sequence, results are significantly improved, which indicates that all models lack an understanding of the human skeleton structure.

Using a method to smooth predicted 3D sequences proved beneficial in most cases, except in the case of VideoPose3D, where it does not seem to improve the predicted sequence, but rather looks like the sequence has already been smoothed directly in VideoPose3D. Figure 16 shows the improvements in pose prediction sequence after applying Kalman smoothing with respect to PA-PC on RI-HJS datasets, and Figure 17 shows the improvements after applying Kalman smoothing and retargeting with respect to PA-MPJPE on the Human3.6M datasets.

The average improvement using 3D predicted sequence smoothing (KSM) is 0.7% for the PCK metric (i.e., 0.57% on Human3.6M and 0.84% on RI-HJS) and 1.4% for the MPJPE metric (i.e., 1.26% on Human3.6M and 1.52% on RI-HJS). Retargeting to standardize both predicted sequence bone lengths and ground truth improved the overall result in all cases. The average improvement using retargeting and smoothing (KSM + RET) is 3.87% for the PCK metric (i.e., 4.04% on Human3.6M and 3.71% on RI-HJS) and 10.1% for the MPJPE metric (i.e., 12.95% on Human3.6M and 7.36% on RI-HJS). Interestingly, retargeting improved EvoSkeleton’s overall score the most on the Human3.6M dataset, but improved VideoPose3D’s overall score the most when evaluated on a custom dataset. This suggests that the models have the potential for performance improvement in constructing valid and consistent poses. Based on these results, we can conclude that of the two-stage models evaluated, the UDP pose + EvoSkeleton proved to be the most robust and stable, achieving the highest overall score on the datasets evaluated.

### 4.2. Analysis of the Pose Estimation Errors

**Figure 18:**
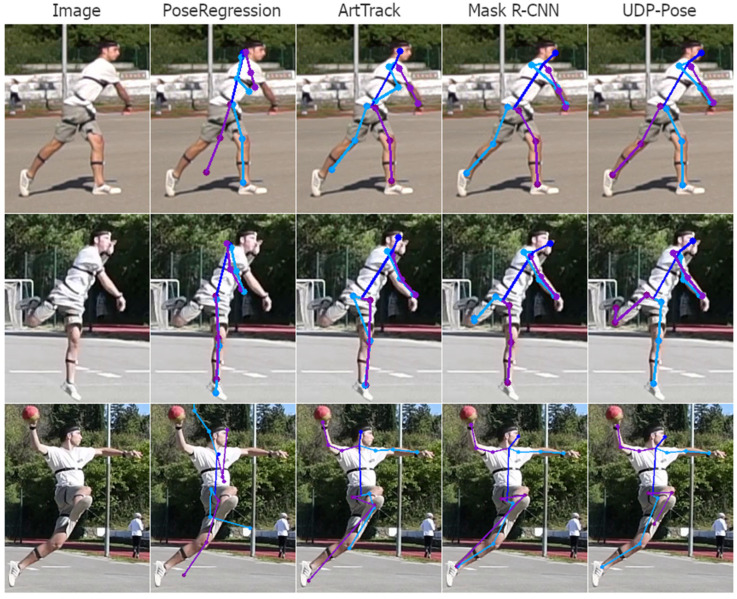
Examples of poor detection of keypoint location that happens mostly because the true keypoint location is occluded or less clear. The right side of the player is coloured purple while the left side of the person is coloured blue. In the first row, where the left elbow and hand are not visible, methods PoseRegression and ArtTrack incorrectly assume the location, while Mask R-CNN and UDP-Pose placed the left elbow and hand on the right elbow and hand of the player. The second row shows a situation where parts are visible but less clear, where all methods fail to detect the left hand, which is close to the head, while methods ArtTrack and Mask R-CNN miss the right foot. The third row shows situations where methods ArtTrack and Mask R-CNN produced invalid human structures by detecting the right foot on the location of the left foot, while the UDP-Pose almost correctly detected the keypoints. PoseRegression generally did not perform well on uncommon poses such as the handball jump-shot.

**Figure 19:**
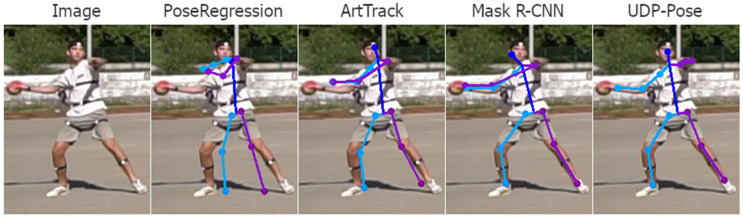
Examples of wrong player side keypoint detection, with an unclear reason for this occurrence. The right side of the player is coloured purple while the left side of the person is coloured blue. While all methods detected almost all keypoints correctly, all methods switched sides of the player, producing an invalid pose. Occurrences of this problem can also be observed on a few keypoints in Figure 18.

Analysing the 3D pose estimation images and predictions, we find that the errors are mainly propagated due to poor 2D pose estimation. Poor predictions occur mainly when one or more joints are occluded. Then the 2D pose estimation model usually assigns the position of the invisible joint to the position of the visible joint on the opposite side. Examples of this problem are shown in Figure 18. Another common problem that is not easily understood or explained is the detection of keypoints on the opposite side of the player. The result is usually a valid human structure but rotated 180 degrees, i.e., the left side is swapped with the right and opposite sides, as shown in Figure 19.

With the goal of reducing errors due to missed detection of visible joints, false detection of visible joints, and invalid pose rotation when switching left and right sides, we trained Mask R-CNN and UDP-Pose on the dataset RI-HJS. Both models were trained on 227 images while evaluation was performed on the rest of the dataset. Both models were trained with a learning rate of 0.001 and an Adam optimizer with 30 epochs. We trained only the Mask R-CNN and UDP-Pose because the PoseRegression and ArtTrack models performed poorly in the previous evaluation in Table 4. The results of the evaluation performed on the test set of the RI-HJS dataset are shown in Table 5.

**Figure 20:**
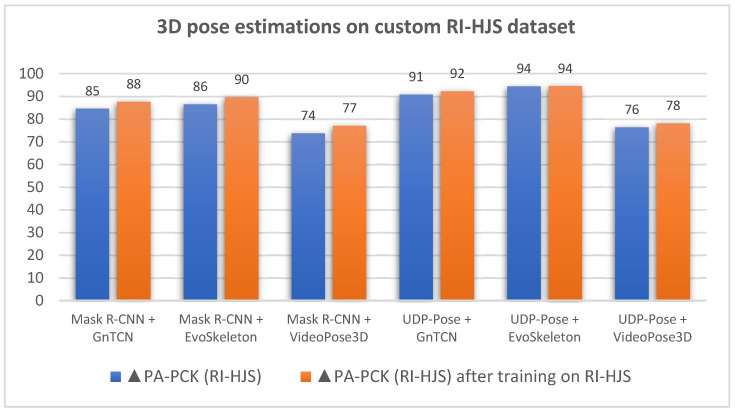
Comparison of pose sequence estimation in terms of PA-PCK on custom RI-HJS datasets before and after additional training of the Mask R-CNN and UDP-Pose models on training part on RI-HJS dataset (higher score is better).

Table 5 shows the results on the test part of the dataset RI-HJS after training the Mask R-CNN and UDP-Pose models on 227 images from the training part of the dataset RI-HJS. The evaluation shows that all two-stage models using models trained on the RI-HJS dataset perform significantly better than models not trained on this dataset. Models using Mask R-CNN show an average improvement of 2.79 on the PA-PCK metric and an average improvement of 0.007 on PA-MPJPE. Models using UDP-Pose show an average improvement of 1.06 on the PA-PCK metric and an average improvement of 0.002 on PA-MPJPE. Even with training, the Mask R-CNN model did not achieve the accuracy of UDP-Pose. In addition, EvoSkeleton appears to be the most robust of the 3D models, providing the best results when paired with both 2D models. A graphical representation of the comparative results before and after training the most successful models Mask R-CNN and UDP-Pose on the training set of RI-HJS is given in Figure 20.

**Figure 21:**
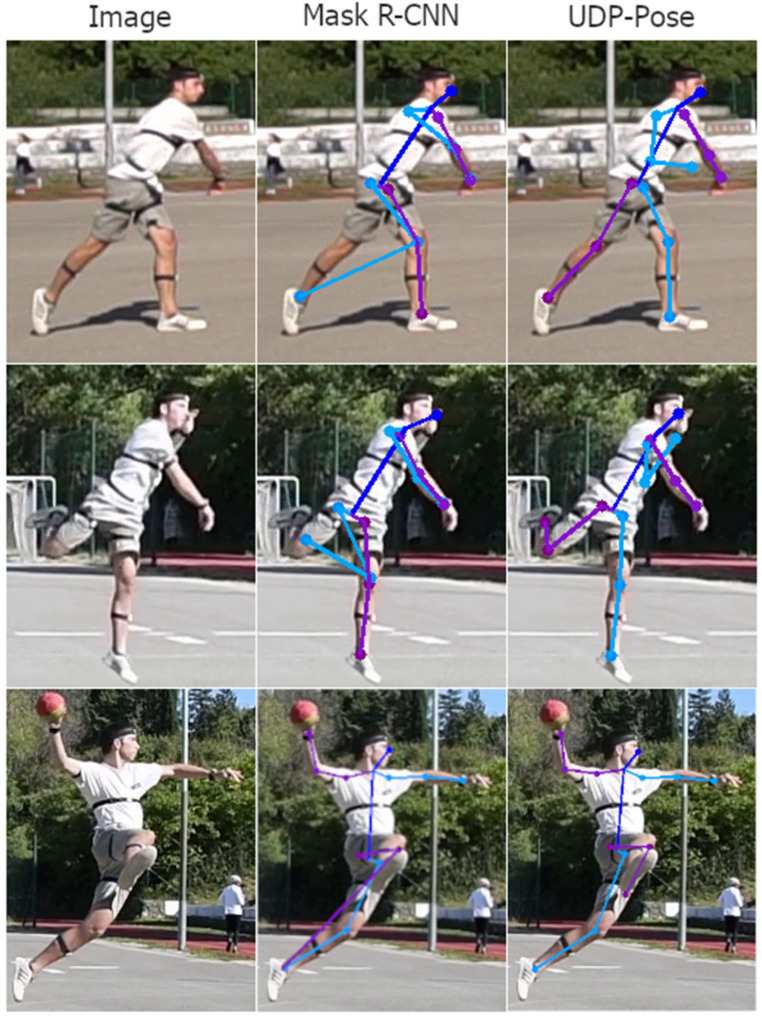
Examples of detection after training on the 227 images of the RI-HJS dataset. The right side of the player is coloured purple while the left side of the person is coloured blue. Untrained models missed detection when the left hand was hidden or less clear, as shown in Figure 18. After training, UDP-Pose successfully detected the left hand on the second row, while on the first row, it made a reasonable guess of the hand position. Mask R-CNN performed worse on both examples after training, wrongly detecting the right knee location on the left knee.

**Figure 22:**
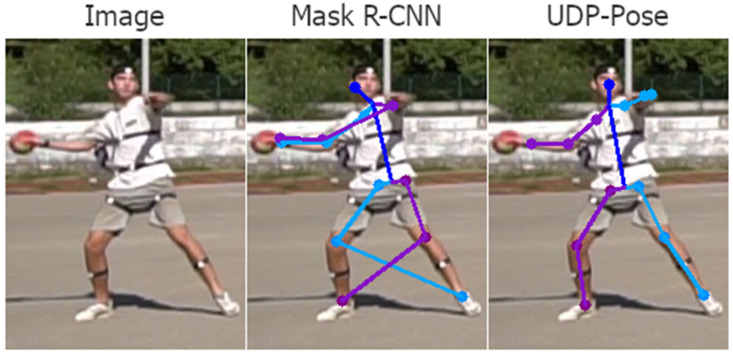
Examples of detection after training on the 227 images of the RI-HJS dataset. The right side of the player is coloured purple while the left side of the person is coloured blue. Untrained models made a mistake and switched the players’ sides, shown in Figure 19. After training, UDP-Pose successfully detected keypoints on the correct sides, while Mask R-CNN did not manage to detect all keypoint sides correctly.

Examples of detections after training the Mask R-CNN and UDP Pose models are shown in Figure 21 and Figure 22. Further comparisons between the trained and untrained 2D models are shown in Supplementary Figures S1–S4.

## 5. Evaluation of Tracking Methods

We chose some well-known and well-performing methods for tracking and at least one method for each methodology. Tracking methods were evaluated against the following datasets: DanceTrack [96], SportsMOT [97], MOT17 [95], and our custom dataset RI-HB-PT.

The goal of this experiment is to evaluate the tracking performance of the methods that provide the best overall results in an unseen environment. We considered the tracking methods CentroidKF (https://github.com/adipandas/multi-object-tracker, accessed on 1 April 2022) [57] (Kalman filter motion tracking), SORT (https://github.com/adipandas/multi-object-tracker, accessed on 1 April 2022) [63] (Kalman filter motion tracking), DeepSORT (https://github.com/abhyantrika/nanonets_object_tracking, accessed on 1 April 2022) [76] (motion and feature tracking), FlowTracker (https://github.com/hitottiez/sdof-tracker, accessed on 1 April 2022) [70] (optical flow motion tracking), and Tracktor++ (https://github.com/phil-bergmann/tracking_wo_bnw, accessed on 1 April 2022) [79] (motion and feature tracking). None of the selected trackers have seen previously tested datasets, so the evaluation was performed on all unseen datasets. The evaluation was performed on the training part of the datasets, since the annotations and ground truths are only available for this part. The final results are shown in Table 6, using the metrics described in Section 3.4.

### Discussion of Tracking Results

Table 6 shows that there is no clear winner that performs best in all datasets or metrics. DeepSORT performs best on the re-identification task and significantly outperforms the other methods on all datasets except RI-HB-PT in terms of IDF1, Identity Switching (IDsw), and Mostly Tracked targets (MT). For the MOTP metric, Tracktor++ and SORT achieve the best overall results, but based on the MOTA metrics, Tracktor++ and DeepSORT share first place. FlowTracker, surprisingly, scores significantly lower than the compared methods, but performs better in datasets where the camera is still and there are fewer entrances and exits from the scene. The CentroidKF and SORT methods rely on the Kalman filter and are very simple, but perform well in certain scenarios. It appears that camera motion strongly influences these methods, as they perform significantly better when the camera is stationary than when it is moving. Table 7 shows the averaged results for all datasets and confirms the observations described earlier. Examples where the models performed poorly are shown in Supplementary Figures S5–S9.

## 6. Conclusions

In this work, we evaluated 12 selected 2-stage models for 3D pose estimation and methods for smoothing and retargeting the sequences. We reported the results and concluded that the application of smoothing and retargeting methods significantly improves the performance of the models. We also evaluated the performance of the two-stage model on a custom dataset to assess its robustness in different/unknown environments. The results of this evaluation are surprising in that most pipelines showed significant performance degradation; only pipelines based on EvoSkeleton had the smallest degradation. However, the UDP-Pose + EvoSkeleton and UDP-Pose + GnTCN models were able to achieve equally high values in both familiar and unfamiliar environments. They achieved an accuracy of correctly estimated body parts of over 90% and a mean joint position error of less than 0.08%, which undoubtedly enables the use of these models for pose estimation in dynamic scenes, such as handball sports.

The greatest performance gain for the models appears to be in constructing good and consistent poses, as smoothing the time series of keypoints over the predicted sequence and retargeting the poses consistently improved the overall score. The improvement in results from smoothing the predicted 3D sequence was seen in the accuracy of the estimated body parts (according to the PCK metric, 0.57% in the Human3.6M dataset and 0.84% in the RI-HJS dataset) and in the reduction of error in the joint position estimation (according to the MPJPE metric, by 1.26% in the Human3.6M dataset and 1.52% in the RI-HJS dataset).

In addition, the retargeting procedure used to normalize the data using the standardized bone length improved the overall score by approximately 4% in all cases in terms of the accuracy of the estimated body parts (i.e., PCK metric 4.04% on Human3.6M and 3.71% on RI-HJS). In addition, the mean error of joint position was reduced by 10% (i.e., according to MPJPE metric 12.95% on Human3.6M and 7.36% on RI-HJS). It is important to note that the performance of top-down pose estimation methods can be affected by object detector performance (e.g., by generating invalid bounding boxes). The detailed performance effects of different object detectors on pose estimation methods are beyond the scope of this work, but may be investigated in future work.

To track the poses of one athlete while acting, poses must be collected throughout the video and mapped on consecutive frames with an algorithm. We selected five state-of-the-art tracking methods and evaluated them against public and user-defined datasets. The main finding after the evaluation is that there is no particular method that performs best in all tested scenarios. However, the DeepSORT method outperforms the rest of the methods in most of the datasets, except for our custom dataset RI-HB-PT, especially in terms of IDF1, Identity switch (IDsw), and Mostly Tracked targets (MT). On the other hand, camera motion seems to strongly affect methods based on the Kalman filter or optical flow, where feature-based tracking methods show their strength. Based on the averaged overall results, we conclude that Tracktor++ and DeepSORT methods provide promising results for tracking people represented by skeletons in dynamic sports scenarios. Therefore, these methods should be considered in the definition of athlete action recognition models.

The experiment has shown that existing state-of-the-art methods for pose estimation already perform satisfactorily and can be used for estimating the poses of a single athlete in individual or team sports. However, for more complex tasks such as tracking more athletes in team sports and comparing athletes’ performances or actions, where multi-object tracking methods are to be used, further research and development of methods are needed to successfully use them in dynamic environments such as sports scenes.

## Figures and Tables

**Table 1:** Comparison of the key differences between methods for 2D pose estimation. A checkmark in column *Structure-aware* represents the methods’ ability to ensure the validity of the human skeleton structure. A checkmark in the column *Use of temporal data* represents whether the method uses previous predictions or other temporal information.

Method	Approach	Human Prediction	Structure-Aware	Use of Temporal Data	Prediction	Type
Toshev and Szegedy [1]	Top-down	Single			Joint	Regression
Carreira et al. [3]	Top-down	Single	**✓**		Joint	Regression
Luvizon et al. [4]	Top-down	Single			Joint	Regression
Sun et al. [5]	Top-down	Single	**✓**		Bone	Regression
Chen and Yuille [6]	Top-down	Single	**✓**		Joint	Heatmap
Newell et al. [7]	Top-down	Single			Joint	Heatmap
Chen et al. [8]	Top-down	Single	**✓**		Joint	Heatmap
Chu et al. [9]	Top-down	Single	**✓**		Joint	Heatmap
He et al. [11]	Top-down	Single			Joint	Heatmap
Radosavovic et al. [13]	Top-down	Single			Joint	Heatmap
Fant et al. [14]	Top-down	Single			Joint	Heatmap
Pishchulin et al. [15]	Bottom-up	Multi			Joint	Regression
Insafutdinov et al. [16,17]	Bottom-up	Multi	**✓**	**✓**	Joint	Heatmap
Cao et al. [18]	Bottom-up	Multi	**✓**		Joint	Heatmap
Newell et al. [19]	Bottom-up	Multi	**✓**		Joint	Heatmap
Huang et al. [20]	Bottom-up	Multi	**✓**		Joint	Heatmap

**Table 2:** Comparison of the key differences between methods for 3D pose estimation. A checkmark in column *Structure-aware* represents the methods’ ability to ensure the validity of the human skeleton structure. A checkmark in the column *Use of temporal data* represents whether the method uses previous predictions or other temporal information. *Image* in column *Input* means that the model predicts directly from the image, while *2D keypoints* means that the model “lifts” the 2D keypoint to the 3D space.

Method	Input	Human Prediction	Structure-Aware	Use of Temporal Data	Prediction	Type
Li and Chan [21]	Image	Single	**✓**		Joint	Regression
Tekin et al. [22]	Image	Single	**✓**		Joint	Regression
Pavlakos et al. [23]	Image	Single			Joint	Heatmap
Martinez et al. [10]	2D keypoints	Single			Joint	Regression
Hossain and Little [24]	2D keypoints	Single		**✓**	Joint	Regression
Pavllo et al. [25]	2D keypoints	Single		**✓**	Joint	Regression
Chen et al. [26]	2D keypoints	Single		**✓**	Joint	Regression
Li et al. [27]	2D keypoints	Single			Joint	Regression

**Table 3:** Training details of the evaluated 2D and 3D pose estimation models. GT in the *Bounding box* column means that the models used ground truth bounding boxes in the training process. The column *2D keypoints* show the 2D pose estimation model, which produced inputs for the training of the 3D pose estimation model.

Model	Dataset	Optimizer	Learning Rate	Epoch	Bounding Box	2D Keypoints
PoseRegression	MPII	RMSProp	0.001	120	GT	-
ArtTrack	MPII	SGD	0.002	20	GT	-
Mask R-CNN	COCO	SGD	0.01	37	GT	-
UDP-Pose	COCO	Adam	0.001	210	GT	-
GnTCN	Human3.6M	Adam	0.001	100	GT	HRNet
EvoSkeleton	Human3.6M	Adam	0.001	200	GT	HRNet
VideoPose3D	Human3.6M	Adam	0.001	80	Mask R-CNN	CPN

**Table 4:** Results of model combinations for 3D pose estimations on the Human3.6M dataset and the custom dataset of players performing handball jump-shot (RI-HJS). The best results are marked in bold. Metrics are computed on the normalized poses using the h-norm described in Section 2.5 and Section 2.6 on 13 keypoints. KSM in the table is shorthand for Kalman smoother, which is applied on the predicted sequence before evaluation, while KSM + RET is shorthand for Kalman smoother applied on the predicted sequence and Retargeting applied both on predicted and ground truth sequences.

Dataset	Models	▲PA-PCK_0.15_	▲PA-PCK_0.15_ + KSM	▲PA-PCK_0.15_ + KSM + RET	▼PA-MPJPE	▼PA-MPJPE + KSM	▼PA-MPJPE + KSM + RET
	PoseRegression + GnTCN	67.742	+1.554	+9.269	0.131	−0.003	−0.021
	PoseRegression + EvoSkeleton	68.257	+0.431	+8.370	0.130	−0.001	−0.019
	PoseRegression + VideoPose3D	69.703	+0.236	+10.065	0.127	0.000	−0.020
	ArtTrack + GnTCN	91.015	+0.769	+3.291	0.067	−0.002	−0.010
	ArtTrack + EvoSkeleton	86.698	+1.888	+6.069	0.079	−0.003	−0.015
Human3.6M	ArtTrack + VideoPose3D	93.056	+0.167	+1.905	0.061	0.000	−0.006
	Mask R-CNN + GnTCN	96.896	+0.210	+0.654	0.049	−0.001	−0.003
	Mask R-CNN + EvoSkeleton	96.275	+0.528	+1.325	0.054	−0.002	−0.007
	Mask R-CNN + VideoPose3D	97.935	−0.068	+0.386	0.045	0.000	−0.002
	UDP-Pose + GnTCN	97.790	+0.140	+0.446	0.045	0.000	−0.003
	UDP-Pose + EvoSkeleton	97.645	+0.271	+0.773	0.049	−0.001	−0.005
	UDP-Pose + VideoPose3D	**98.023**	−0.075	+0.345	**0.044**	0.000	−0.002
	PoseRegression + GnTCN	60.381	+0.283	+2.057	0.150	−0.001	−0.008
	PoseRegression + EvoSkeleton	62.475	+0.357	+2.158	0.144	−0.001	−0.006
	PoseRegression + VideoPose3D	58.784	+0.290	+5.029	0.154	0.000	−0.013
	ArtTrack + GnTCN	80.310	+0.864	+1.398	0.106	−0.002	−0.006
	ArtTrack + EvoSkeleton	80.549	+2.079	+2.501	0.107	−0.006	−0.010
RI-HJS	ArtTrack + VideoPose3D	59.736	−0.027	+8.668	0.151	0.000	−0.020
	Mask R-CNN + GnTCN	84.545	+0.771	+1.135	0.098	−0.002	−0.005
	Mask R-CNN + EvoSkeleton	86.485	+2.132	+2.415	0.094	−0.006	−0.010
	Mask R-CNN + VideoPose3D	73.718	−0.152	+3.084	0.124	0.000	−0.008
	UDP-Pose + GnTCN	90.797	+0.551	+1.370	0.083	−0.001	−0.004
	UDP-Pose + EvoSkeleton	**94.436**	+0.695	+1.009	**0.074**	−0.002	−0.005
	UDP-Pose + VideoPose3D	76.357	−0.232	+2.916	0.117	−0.000	−0.007

**Table 5:** Results of model combinations for 3D pose estimations on the custom dataset of players performing handball jump-shot (RI-HJS). The best results are marked in bold. Models Mask R-CNN and UDP-Pose were trained on 227 images from the RI-HJS dataset, while evaluation was performed on the rest of the dataset. Metrics are computed on the normalized poses using the h-norm described in Section 2.5 and Section 2.6 on 13 keypoints. KSM in the table is shorthand for Kalman smoother that is applied on the predicted sequence before evaluation, while KSM + RET is shorthand for Kalman smoother applied on the predicted sequence and Retargeting applied both on predicted and ground truth sequences.

Dataset	Models	▲PA-PCK_0.15_	▲PA-PCK_0.15_ + KSM	▲PA-PCK_0.15_ + KSM + RET	▼PA-MPJPE	▼PA-MPJPE + KSM	▼PA-MPJPE + KSM + RET
	Mask R-CNN + GnTCN	87.574	+0.644	+0.970	0.090	−0.002	−0.004
	Mask R-CNN + EvoSkeleton	89.562	+1.875	+2.004	0.086	−0.006	−0.009
RI-HJS	Mask R-CNN + VideoPose3D	76.970	−0.196	+2.258	0.118	0.000	−0.007
	UDP-Pose + GnTCN	92.205	+0.486	+0.974	0.080	−0.001	−0.003
	UDP-Pose + EvoSkeleton	**94.462**	+0.537	+0.303	**0.073**	−0.002	−0.003
	UDP-Pose + VideoPose3D	78.122	−0.267	+2.053	0.115	−0.000	−0.006

**Table 6:** Results of tracking methods on DanceTrack, SportsMOT, MOT17, and a custom RI-HB-PT dataset of players practicing various handball actions during a practice session. The best results according each metrics are marked in bold. Metrics are computed as described in Section 3.4.

Dataset	Models	▲MOTA	▲MOTP	▲IDF1	▼IDsw	▲Recall	▲Precision	▲MT	▼ML
	CentroidKF	**76.9**	0.201	7.9	47,550	95.3	95.3	409	**0**
	SORT	27.5	**0.313**	11.7	16,052	66.1	66.1	20	**0**
DanceTrack	DeepSORT	68.0	0.160	**43.0**	**4717**	**97.6**	77.5	**418**	**0**
	FlowTracker	38.1	0.262	13.5	10,448	66.4	72.4	59	1
	Tracktor++	67.4	0.262	29.4	18,255	75.1	**96.9**	166	**0**
	CentroidKF	15.4	0.247	5.9	43,469	64.7	64.7	133	**0**
	SORT	15.6	0.254	6.2	48,186	65.5	65.5	4	**0**
SportsMOT	DeepSORT	**79.9**	0.149	**63.7**	**2939**	**99.2**	84.4	**635**	**0**
	FlowTracker	25.4	0.281	12.2	9873	62.6	64.7	16	3
	Tracktor++	64.6	**0.297**	42.9	7949	78.0	**89.8**	298	**0**
	CentroidKF	60.7	0.140	49.0	11,200	83.1	83.1	366	26
	SORT	56.5	0.159	52.4	9909	80.7	80.7	220	**2**
MOT17	DeepSORT	**71.0**	0.062	**70.9**	**1159**	**90.5**	90.5	**664**	24
	FlowTracker	37.1	0.156	38.2	2093	47.4	83.6	162	310
	Tracktor++	64.8	**0.258**	64.8	3263	73.4	**91.3**	356	115
	CentroidKF	69.4	0.231	19.3	9912	87.0	87.0	310	15
	SORT	49.0	**0.261**	21.4	6299	76.0	76.0	225	16
RI-HB-PT	DeepSORT	70.2	0.063	38.7	2276	**99.7**	77.8	**353**	**4**
	FlowTracker	68.0	0.222	16.6	3770	84.6	85.1	125	145
	Tracktor++	**92.4**	0.202	**49.6**	**1346**	94.8	**98.1**	176	137

**Table 7:** Averaged results of trackers across all datasets (DanceTrack, SportsMOT, MOT17, RI-HB-PT), i.e., averaged results from Table 6. The best results according each metrics are marked in bold.

Models	▲MOTA	▲MOTP	▲IDF1	▼IDsw	▲Recall	▲Precision	▲MT	▼ML
CentroidKF	55.60	0.204	20.52	28,057	82.52	82.52	304	10
SORT	37.15	0.246	22.92	20,111	72.07	72.07	117	**4**
DeepSORT	72.15	0.108	**54.07**	**2772**	**96.75**	80.60	**517**	7
FlowTracker	42.15	0.230	20.12	6546	65.25	76.45	88	115
Tracktor++	**72.30**	**0.254**	46.67	7703	80.32	**93.52**	249	63

## Data Availability

The data presented in this study are available on request from the corresponding author. The data are not publicly available due to privacy restrictions.

## Supplementary Materials

The following are available online at https://www.mdpi.com/article/10.3390/jimaging8110308/s1, Figure S1: Example of 3D sequences produced by three 3D HPE models and Mask R-CNN, Figure S2: Example of 3D sequences produced by three 3D HPE models and trained Mask R-CNN on RI-HJS dataset, Figure S3: Example of 3D sequences produced by three 3D HPE models and UDP-Pose, Figure S4: Example of 3D sequences produced by three 3D HPE models and trained UDP-Pose on RI-HJS dataset, Figure S5: Example of scenario where CentroidKF tracker performs poorly, Figure S6: Example of scenario where SORT tracker performs poorly, Figure S7: Example of scenario where FlowTracker tracker performs poorly, Figure S8: Example of scenario where DeepSORT tracker performs poorly, Figure S9: Example of scenario where Tracktor++ tracker performs poorly.
