# Supplementary material for: 3D Pose Estimation and Tracking in Handball Actions Using a Monocular Camera
Source: J Imaging. 2022 Nov 10;8(11):308. doi: 10.3390/jimaging8110308 (PMC9693185; doi:10.3390/jimaging8110308)
Supplement: Supplementary file 1 [file jimaging-08-00308-s001.zip › jimaging-1893862-supplementary.pdf]

Supplementary Materials

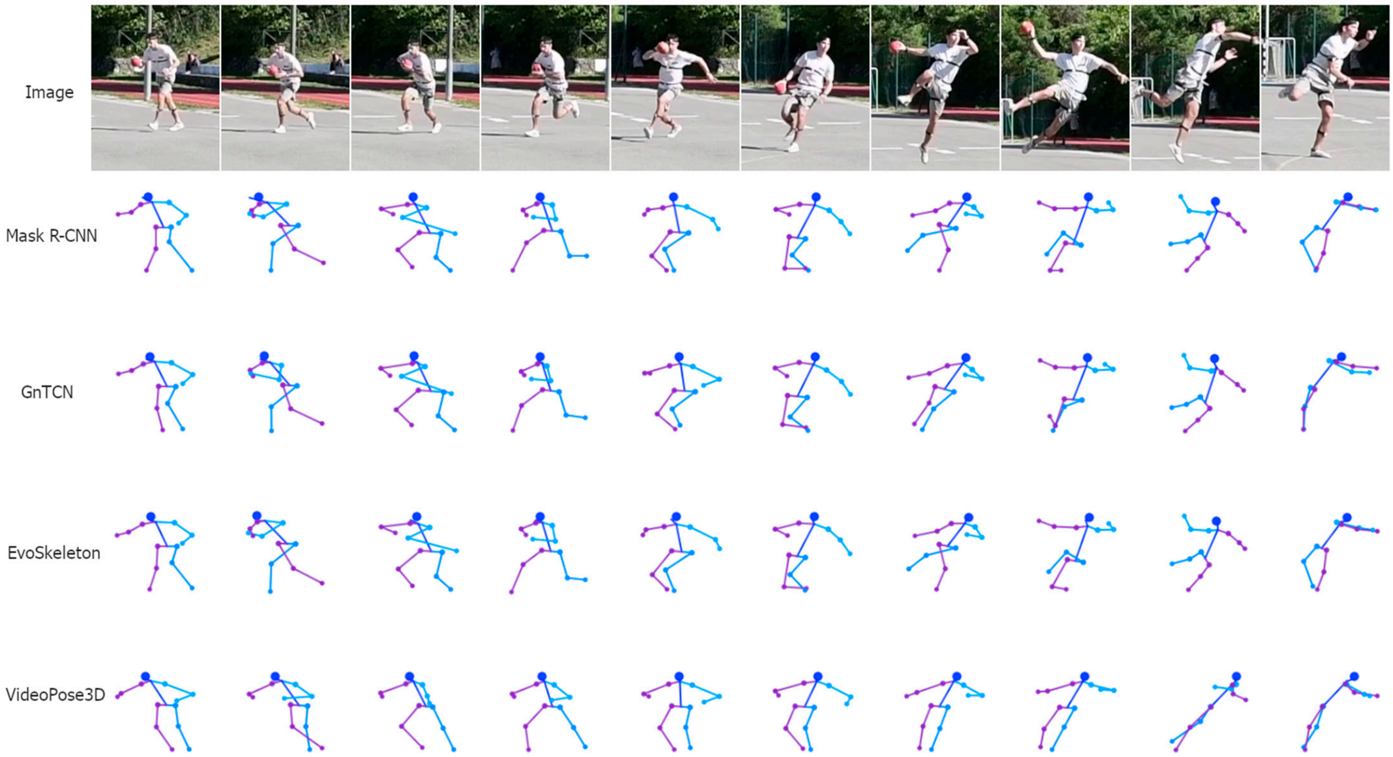

Figure S1. 3D pose estimation by models GnTCN, EvoSkeleton and VideoPose3D using 2D keypoints produced by **Mask R-CNN**. The right side of the player is colored purple while the left side of the person is colored blue.

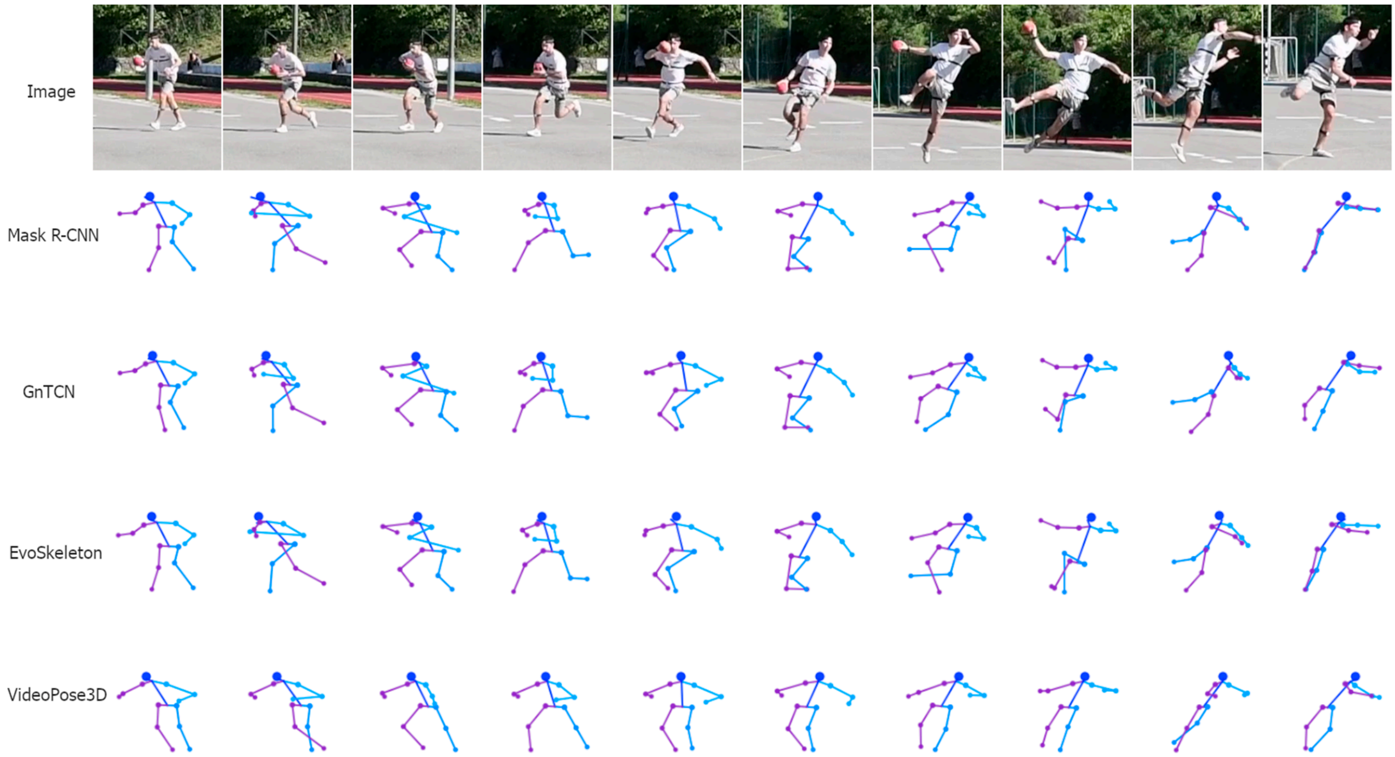

Figure S2. 3D pose estimation by models GnTCN, EvoSkeleton and VideoPose3D using 2D keypoints produced by trained **Mask R-CNN** model on 227 images from RI-HJS dataset. The right side of the player is colored purple while the left side of the person is colored blue.

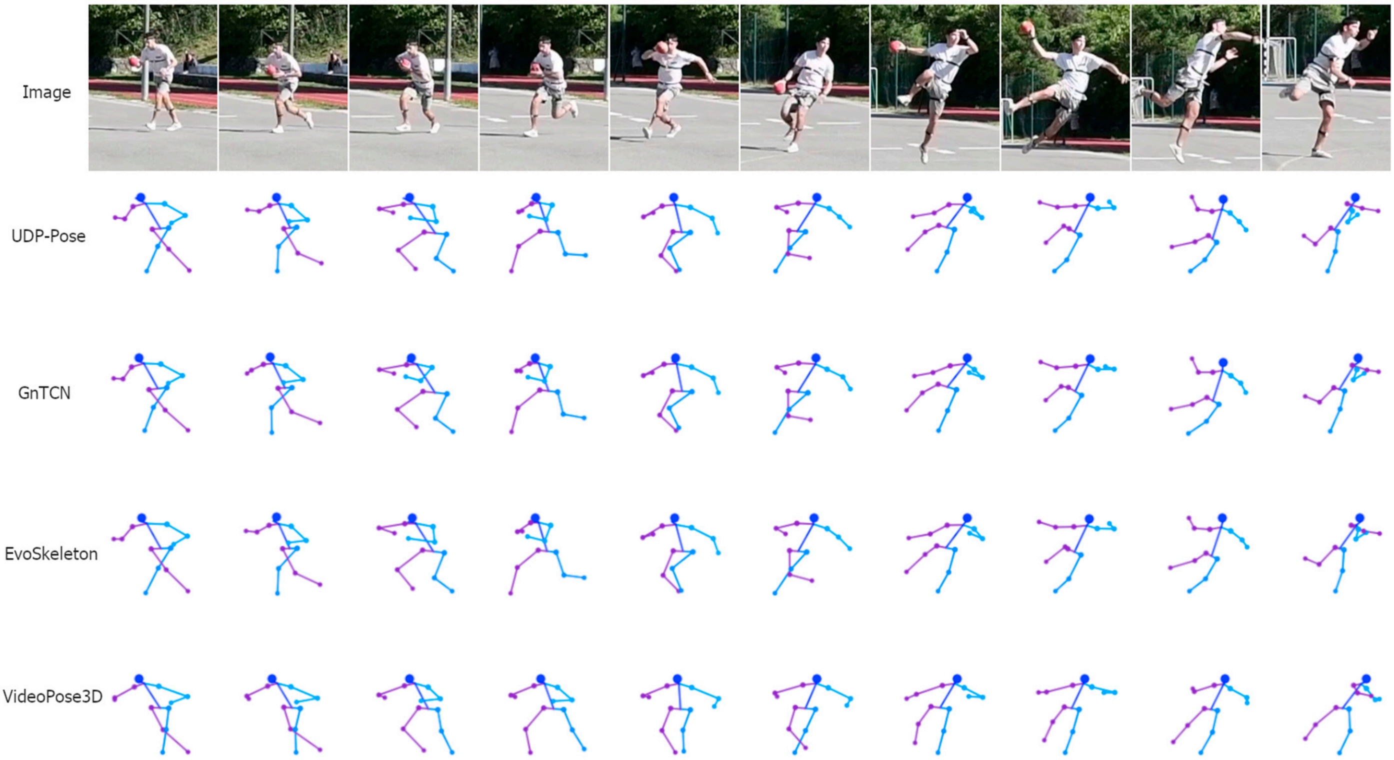

Figure S3. 3D pose estimation by models GnTCN, EvoSkeleton and VideoPose3D using 2D keypoints produced by **UDP-Pose**. The right side of the player is colored purple while the left side of the person is colored blue.

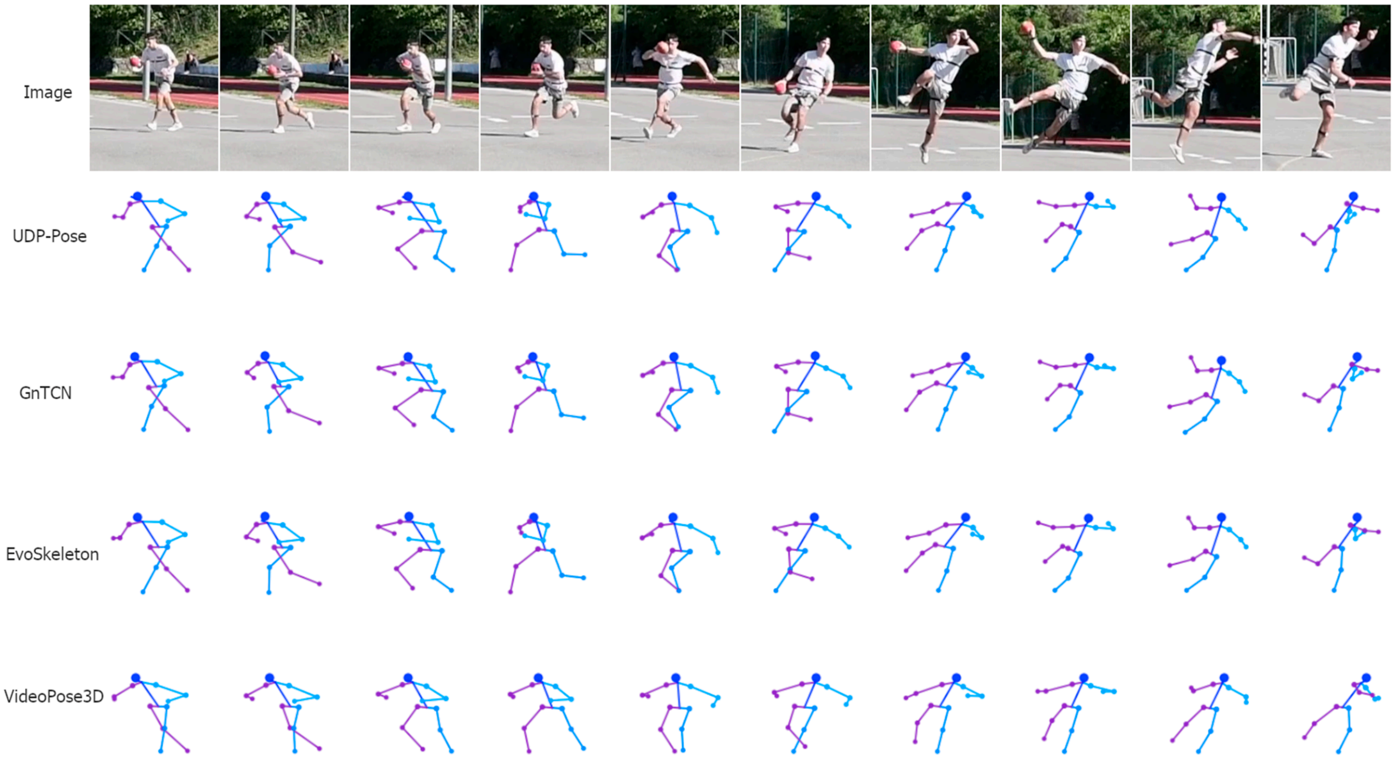

Figure S4. 3D pose estimation by models GnTCN, EvoSkeleton and VideoPose3D using 2D keypoints produced by trained **UDP-Pose** model on 227 images from RI-HJS dataset. The right side of the player is colored purple while the left side of the person is colored blue.

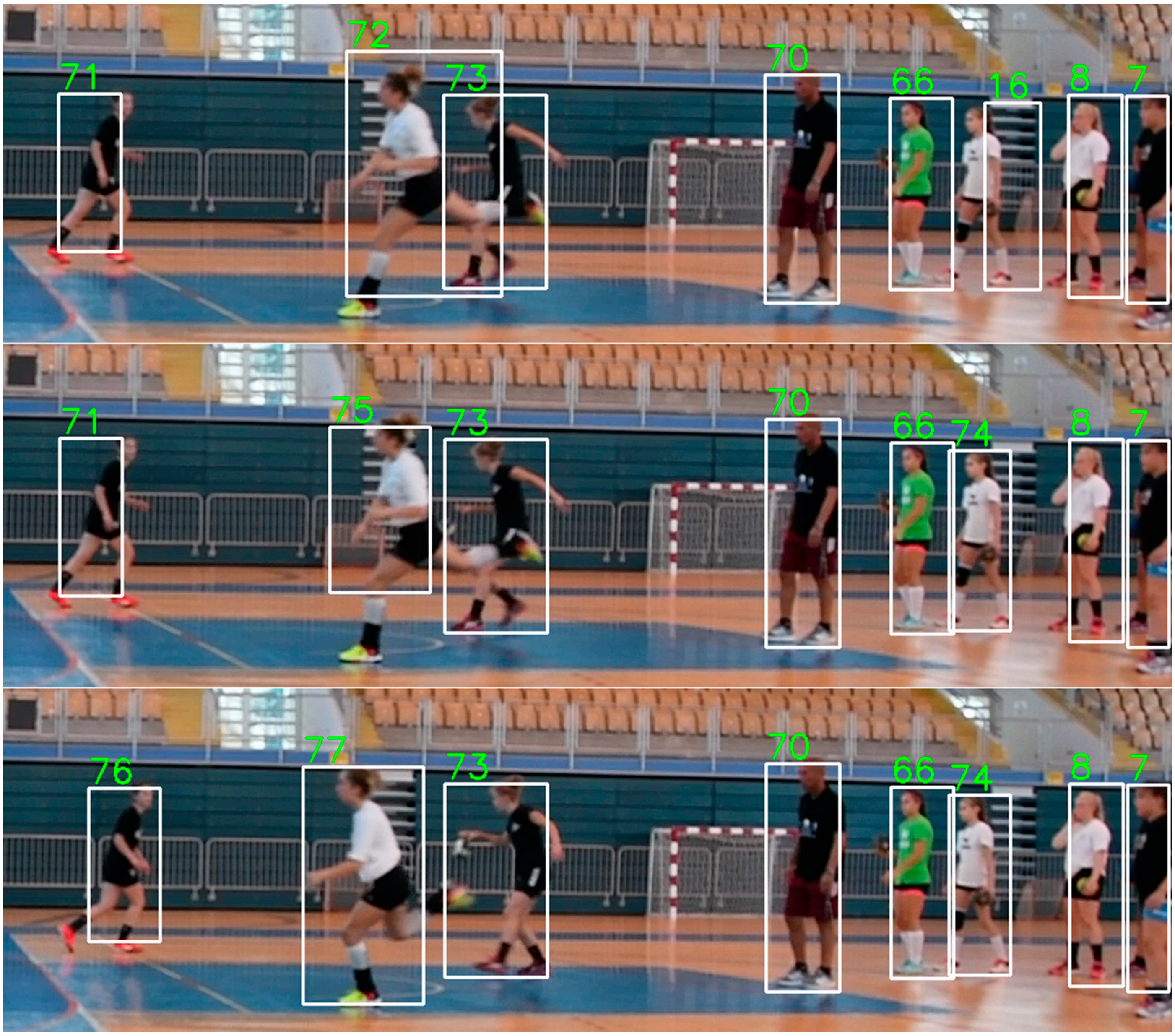

Figure S5. Example of scenario where **CentroidKF** tracker performs poorly. On the first row, the bounding box for the player with the id of 72 is significantly bigger than the following bounding box on the second row. The difference between the centers of the two bounding boxes is large enough to force the algorithm to assign a new id of 75 to the player with the id of 72. The same problem occurs in the third row, where the bounding box of the same player is significantly larger than in the second row, which again produces a new id of 77. Similarly, the bounding box for the player with the id of 71 in the first and second rows stays in a similar position during the player's movement to the right. In the third row, the bounding box is aligned with the player, and the algorithm assigns a new id again. The algorithm is highly dependent on the correct and consistent bounding box detection because even changes in sizes in the bounding box across two consecutive frames can cause an error in tracking.

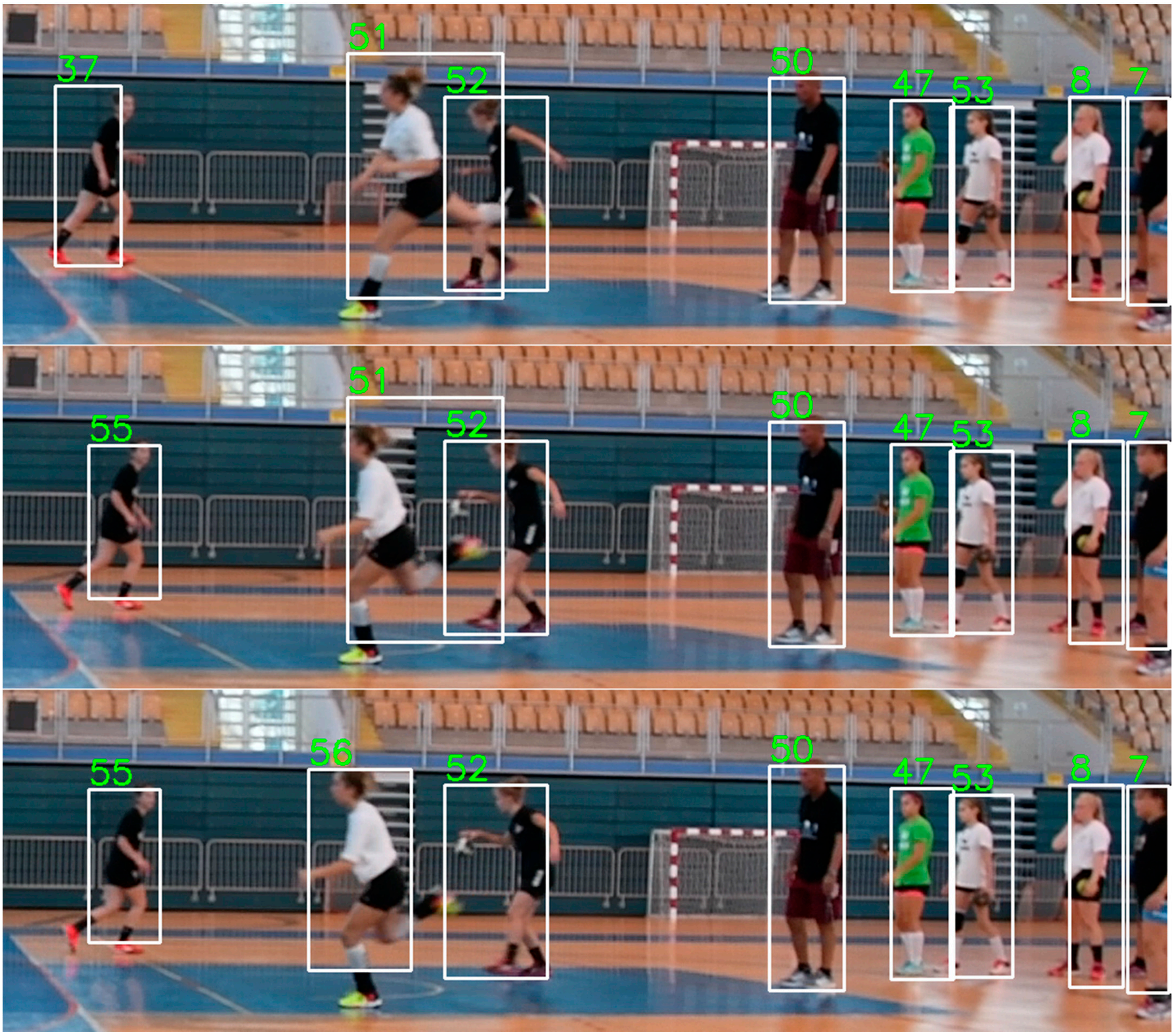

Figure S6. Example of scenario where **SORT** tracker performs poorly. On the first and second rows, the bounding box for the player with the id of 71 is larger and better surrounds the player, but on the third row, it significantly reduces its size, which is enough for the SORT tracker to assign the player with a new id. A similar problem occurs with the player with the id of 37 in the first row, wherein the second row changes the position significantly enough for SORT to assign the player a new id. The algorithm is highly dependent on the correct and consistent bounding box detection because even changes in sizes in the bounding box across two consecutive frames can cause an error in tracking.

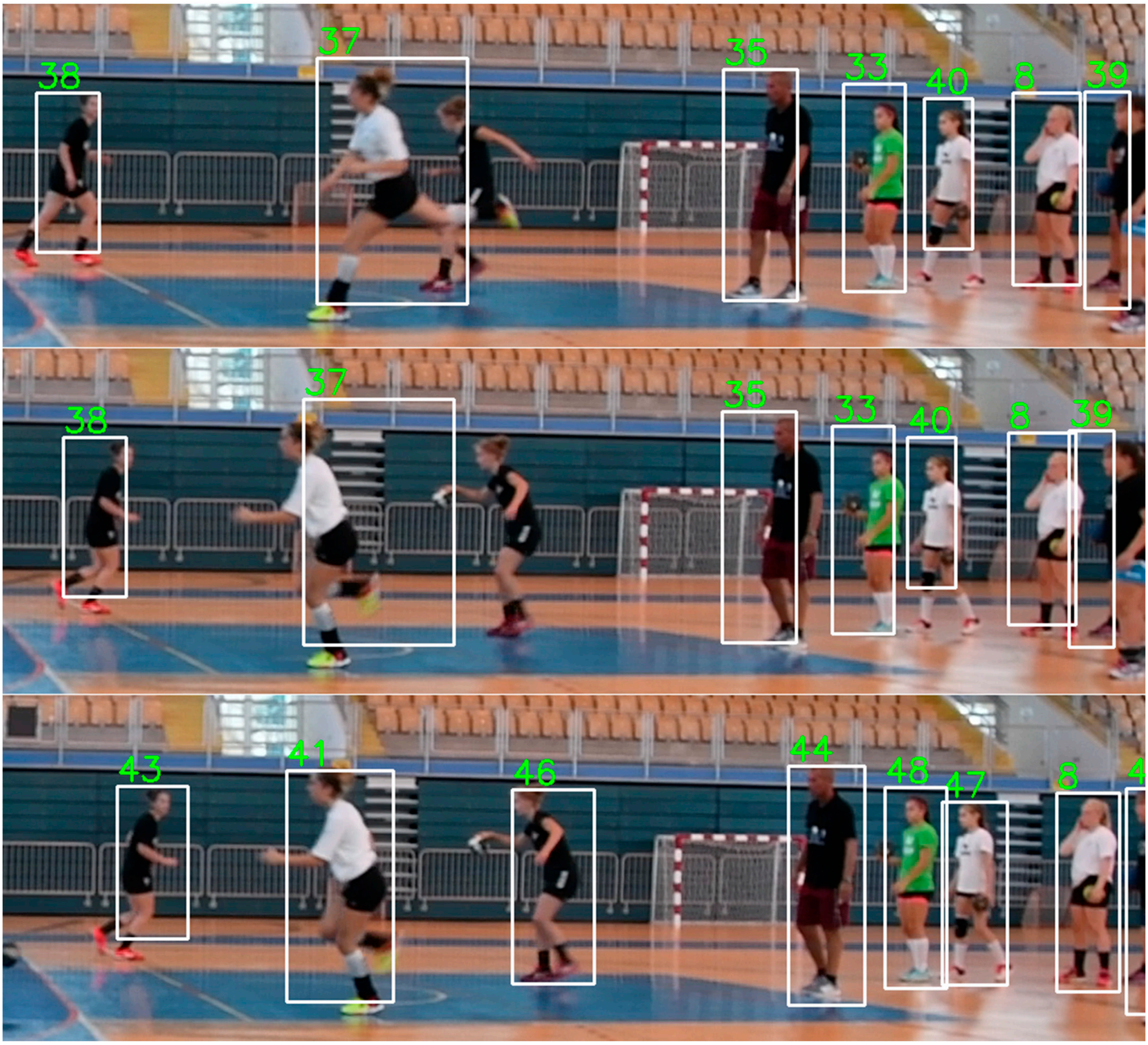

Figure S7. Example of scenario where **FlowTracker** tracker performs poorly. Generally, whenever a bounding box is significantly moved or changes its size, the FlowTracker assigns the player a new id. The FlowTracker also produces False Negatives, as is an example of the player with the id of 46 in the third row that is not tracked in previous rows. The algorithm is highly dependent on the correct and consistent bounding box detection because even changes in sizes in the bounding box across two consecutive frames can cause an error in tracking.

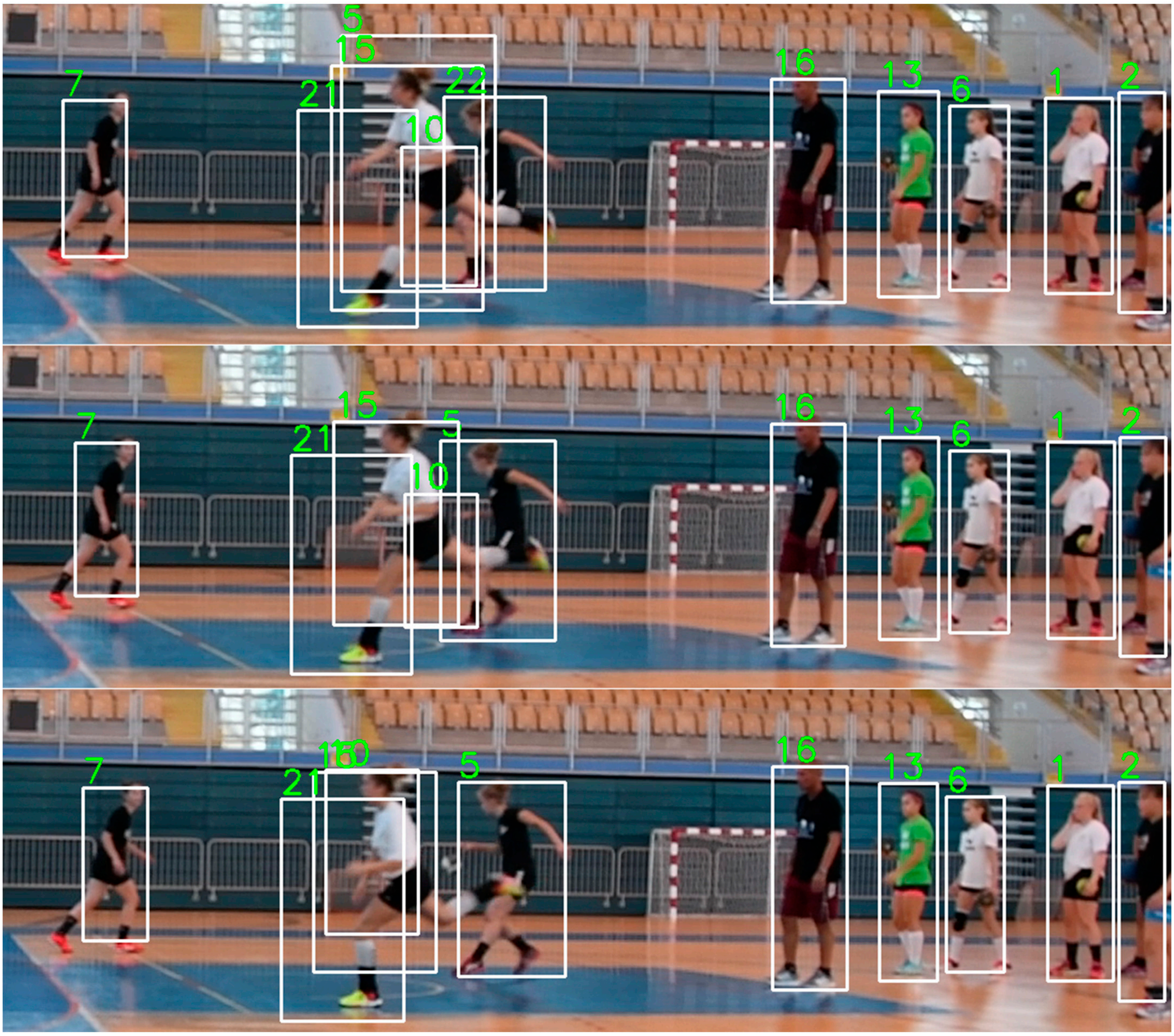

Figure S8. Example of scenario where **DeepSORT** tracker performs poorly. DeepSORT continues to track players that do not exist, producing a lot of False Positives, but even in that chaos, it continues to track the player with the id of 15 while it passes in front of the other player. However, the player with the id of 22 in the first row changes the id to 5 in the following two rows, meaning that DeepSORT did not manage to capture a robust enough feature representation that recognizes the object even when partially occluded. DeepSORT is less sensitive to poor consistency of bounding box detection than other methods because it uses feature-based tracking that helps to retain object id even when the bounding box changes size and shape.

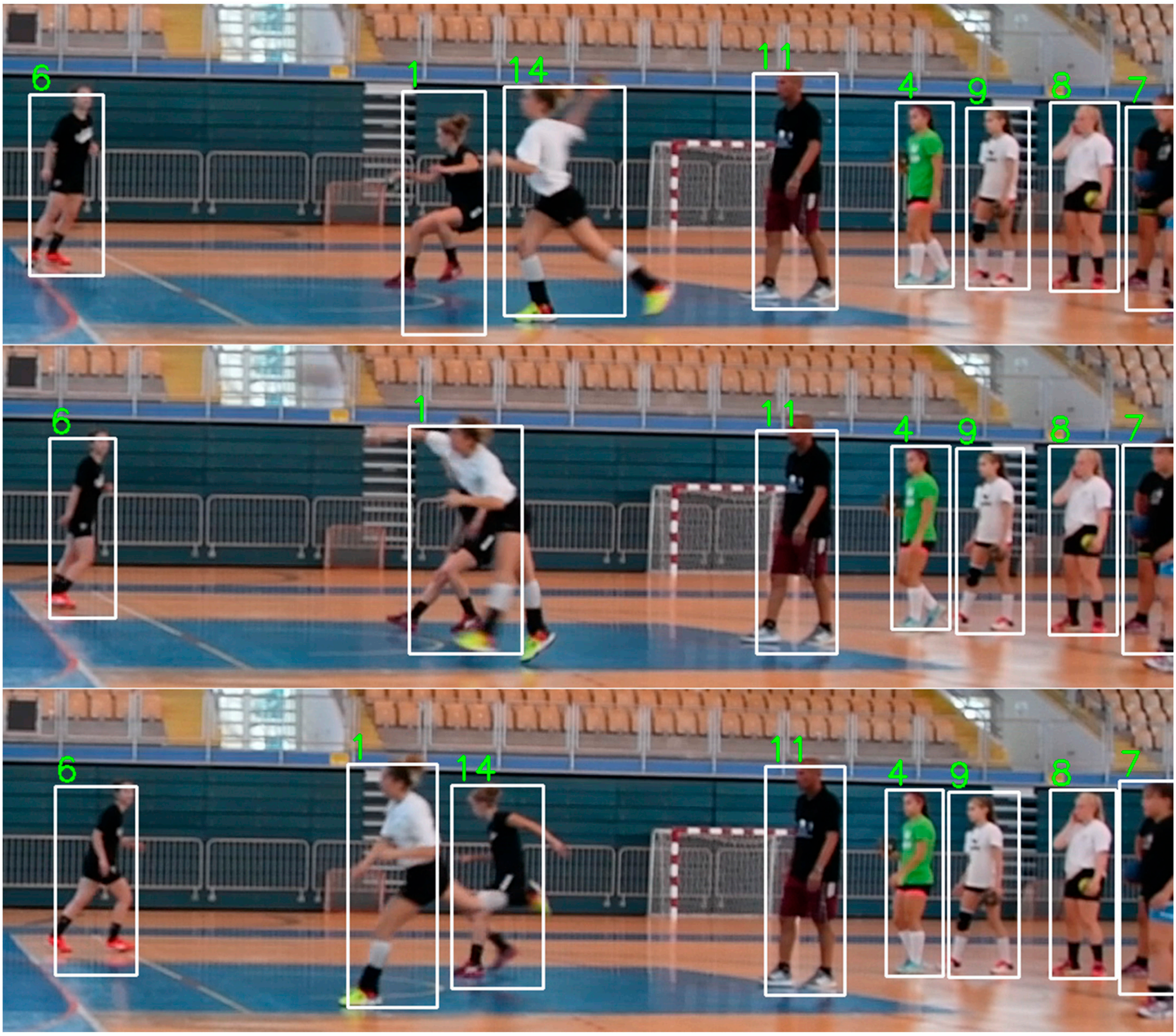

Figure S9. Example of scenario where **Tracktor++** tracker performs poorly. Tracktor++ performs well when tracking the players while running across the field but struggles when the players occlude each other. In this example, Tracktor++ switched the ids of players with ids 1 and 14 after one player passed in front of the other. Given that Tracktor++ combines motion-based and feature-based tracking, it is unexpected that this kind of error occurs when players have different appearances and motions. Tracktor++ is less sensitive to poor consistency of bounding box detection than other methods because it uses feature-based tracking that helps to retain object id even when the bounding box changes size and shape.
